# Manganese Oxide Catalysts for Lithium–Oxygen Batteries: Structures, Mechanisms, and Reaction Pathway Engineering

**DOI:** 10.1002/smll.74270

**Published:** 2026-06-27

**Authors:** Ruiqin Peng, Linna Dai, Karol Viviana Mejia‐Centeno, Malik Dilshad Khan, Guifang Zeng, Yanhong Tian, Andreu Cabot, Qing Sun

**Affiliations:** ^1^ School of Intelligence Engineering Shandong Management University Jinan China; ^2^ School of Science Hubei University of Technology Wuhan China; ^3^ Catalonia Institute for Energy Research (IREC) Barcelona Spain; ^4^ Faculty of Chemistry University of Barcelona Barcelona Spain; ^5^ State Key Laboratory of Precision Welding & Joining of Materials and Structures Harbin Institute of Technology Harbin China; ^6^ ICREA Pg. Lluis Companys Barcelona Spain

**Keywords:** catalysts, lithium‐oxygen batteries, manganese oxides, oxygen evolution reaction, oxygen reduction reaction

## Abstract

The growing demand for high‐specific‐energy storage has revived interest in lithium‐oxygen batteries (LOBs), whose theoretical energy density far exceeds that of conventional lithium‐ion battery systems. However, their practical use is limited by sluggish reaction kinetics, parasitic reactions, high overpotentials, and the buildup of insulating lithium peroxide, all of which hinder reversibility and cycling stability. Manganese oxides (MnO_x_) have emerged as promising cathode catalysts because of their abundance, low cost, tunable oxidation states, and diverse structural features. Their tunnel, layered, and spinel architecture provide adaptable environments for O_2_ reduction and evolution, Li^+^ transport, and the conversion of reaction intermediates. Recent progress demonstrates that structural engineering of MnO_x_, through pore‐structure tuning, surface‐site modulation, defect introduction, heteroatom doping, and composite fabrication, can significantly optimize lithium peroxide formation and decomposition. These strategies regulate catalyst electronic structures and reaction pathways, thereby influencing Li_2_O_2_ formation/decomposition behavior, reducing side reactions, lowering polarization, and enhancing catalytic activity. This review summarizes recent advances in MnO_x_‐based catalysts for non‐aqueous LOBs, emphasizing structure‐activity relationships and mechanistic understanding. By outlining remaining challenges and key design guidelines, we aim to support the rational development of next‐generation catalysts for practical LOB deployment.

## Graphical abstract

How do the morphologies, crystal structures, and electronic properties of manganese oxides influence oxygen reaction pathways in lithium–oxygen batteries (LOBs)? This review systematically summarizes structure‐dependent catalytic mechanisms and recent engineering strategies, highlighting how defect engineering, compositional modulation, and reaction‐pathway regulation enhance reversibility and advance practical high‐energy LOB systems.

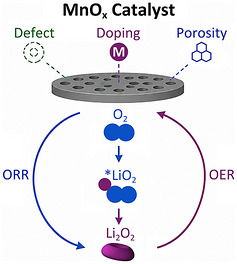

## 1 Introduction

With the accelerating demand for high‐specific‐energy storage systems driven by portable electronics [1, 2, 3, 4, 5], electric vehicles [6, 7, 8, 9], and large‐scale grid applications [10, 11, 12, 13], emerging rechargeable battery technologies that transcend the energy‐density limit of conventional lithium batteries have attracted extensive attention [14, 15, 16, 17]. Among these candidates, lithium‐oxygen batteries (LOBs), employing metallic Li as the anode and O_2_ as the cathodic reactant, stand out due to their gasoline‐comparable theoretical energy density [18]. Nevertheless, the practical implementation of LOBs remains severely constrained by several intrinsic bottlenecks. The charge/discharge process involves heterogeneous reactions across solid–liquid–gas interfaces. The primary discharge product, Li_2_O_2_, is insoluble and electronically insulating in organic electrolytes [19]. Consequently, uncontrolled accumulation of Li_2_O_2_ readily blocks cathode pores, passivates reaction interfaces, elevates polarization, and drastically shortens cycle life [20]. These challenges collectively undermine the energy efficiency and durability required for practical deployment, thereby hindering further advancement of LOB systems [21].

Since Littauer et al. first proposed the concept of LOBs in 1976 [22], their architecture and reaction environment have undergone continuous refinement. Early aqueous configurations suffered from severe safety issues due to the vigorous reactivity between metallic Li and water, limiting their applicability. It was not until 1996 that Abraham et al. introduced a rechargeable non‐aqueous LOB system employing a solid polymer electrolyte membrane [23], marking a major milestone in the field. Subsequently, in 2006, the Bruce group employed in situ mass spectrometry to confirm the reversible decomposition of Li_2_O_2_ into Li and O_2_ during charging [24], a pivotal mechanistic revelation that catalyzed the rapid development of high‐performance, reversible LOBs.

Over the past decade, substantial efforts have been dedicated to enhancing reaction kinetics, mitigating parasitic pathways and improving cycling stability, with notable breakthroughs in cathode catalyst design [25]. Reported catalyst systems include carbon‐based materials, noble‐metal catalysts, transition metal (TM) oxides, and soluble redox mediators (RMs). Yet, each category exhibits inherent limitations [26], carbon materials are prone to parasitic reactions at high potentials; noble metals, despite their superb catalytic activity, suffer from high cost and scarcity; and many TM oxides display limited conductivity and structural instability. Therefore, designing low‐cost, highly active, and structurally tunable cathode catalysts remains a central scientific challenge for the practical realization of LOBs [27].

Among various catalyst candidates, TM oxide, particularly manganese oxide (MnO_x_), have emerged as highly compelling [28]. MnO_x_ benefit from natural abundance, intrinsically favorable oxygen evolution reaction/oxygen reduction reaction (OER/ORR) activity, and diverse oxidation states and polymorphic structures. Distinct MnO_2_ polymorphs (α, β, γ, δ, λ, etc.) offer various tunnel and layered frameworks that facilitate the transport and transformation of O_2_, Li^+^, and Li_x_O_y_ intermediates [29]. Moreover, the redox flexibility of Mn enables activity enhancement through defect engineering, heteroatom modulation, and rational exposure of specific crystal facets [30]. Previous studies have demonstrated that constructing hierarchical porous structures, tailoring surface active sites, and optimizing interfacial reaction pathways can substantially improve the reversible formation/decomposition of Li_2_O_2_ [31]. These modifications suppress side reactions, enhance discharge capacity, reduce overpotential, and improve cycling stability.

Against this backdrop, this review systematically examines recent advances in the structural design and performance modulation of MnO_x_‐based cathode catalysts for LOBs. Emphasis is placed on pore‐architecture engineering, active‐site regulation, crystal‐phase and defect manipulation, and multi‐component synergistic strategies. Furthermore, we discuss the mechanistic roles of Mn‐based catalysts in facilitating the reversible formation of Li_2_O_2_, aiming to provide new perspectives and theoretical guidance for the development of efficient, low‐cost cathode catalysts for next‐generation LOB systems.

## 2 Classification, Challenges, and Reaction Mechanisms of LOBs

**FIGURE 1:**
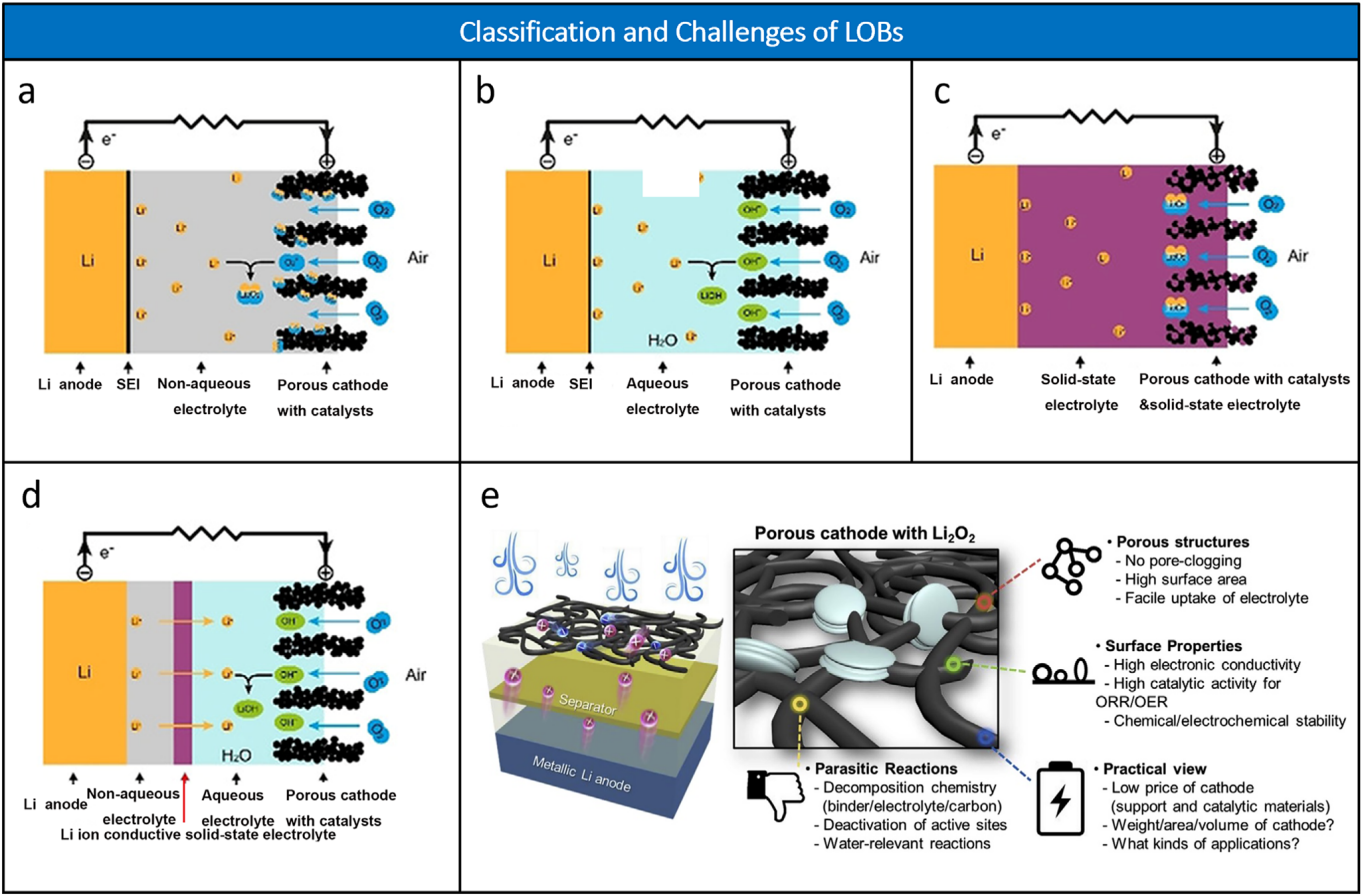
(a–d) Schematic diagrams of LOBs with different electrolytes: non‐aqueous (a), aqueous (b), solid‐state (c), and hybrid (d). Reproduced with permission [32]. Copyright 2019, Wiley. e) Schematic diagram of LOB configuration and practical problems in cathode materials. Reproduced with permission [33]. Copyright 2020, Elsevier.

A LOB typically consists of four major components: a porous O_2_ cathode, a metallic Li anode, an electrolyte, and a waterproof‐breathable membrane (the latter can be omitted under a dry O_2_ atmosphere). According to the type and physical state of the electrolyte layer, LOBs can be categorized into four configurations (Figure 1a–d) [32, 34]: non‐aqueous systems [35], aqueous systems [36], solid‐state systems [37], and hybrid aqueous/non‐aqueous systems [38].

The charge/discharge process proceeds as follows: during discharge, O_2_ from the environment diffuses into the porous cathode and is activated and reduced on the catalytic surface. Meanwhile, Li metal at the anode is oxidized to Li^+^, which migrates through the electrolyte [39]. At the gas‐liquid‐solid three‐phase interface, comprising O_2_, electrolyte, and the catalyst surface, Li^+^ reacts with reduced O species to form solid discharge products that are insoluble and electronically insulating in organic electrolytes [40]. During charging, these solid products are oxidatively decomposed under the catalysis of the cathode material, regenerating Li^+^ and releasing O_2_. Thus, the entire electrochemical process occurs at the cathode side, with Li^+^ shuttling through the electrolyte to reach the reaction sites.

The electrochemical reaction mechanisms of the various LOB configurations are summarized in Table 1. In aqueous systems, direct contact between metallic Li and the aqueous electrolyte leads to spontaneous reduction reactions, posing severe safety hazards [41]. In hybrid aqueous/non‐aqueous systems, an organic electrolyte is employed on the Li side while an aqueous electrolyte is used at the O_2_ electrode. A solid electrolyte layer (Li^+^ conducting ceramic membrane) is inserted between them to block direct contact while enabling Li^+^ transport. This design protects the Li metal and mitigates the continuous accumulation of solid Li_2_O_2_ on the O_2_ electrode. However, the solid electrolyte layer is chemically unstable in strongly acidic or alkaline environments, and significant differences in Li^+^ diffusion kinetics across multiple electrolyte phases further limit practical performance.

**TABLE 1:** Electrochemical reaction mechanisms in LOBs with different electrolytes.

Reaction	Non‐aqueous system	Aqueous and hybrid systems	Solid‐state system
Cathode reaction	O_2_ + 2Li^+^ + 2e^−^ → Li_2_O_2_	O_2_ + 2H_2_O + 4e^−^ → 4OH^−^ (Alkaline) O_2_ + 4e^−^ + 4H^+^ → 2H_2_O (Acidic)	O_2_ + 2Li^+^ + 2e^−^ → Li_2_O_2_
Anode reaction	Li → Li^+^ + e^−^		
Overall reaction	2Li + O_2_ → Li_2_O_2_ (*E*_0_ = 2.96 V vs. Li/Li^+^)	O_2_ + 2H_2_O + 4Li ↔ 4Li^+^ + 4OH^−^ (*E*_0_ = 3.43 V vs. Li/Li^+^, Alkaline) O_2_ + 4H^+^ + 4Li ↔ 4Li^+^ + 2H_2_O (*E*_0_ = 4.26 V vs. Li/Li^+^, Acidic)	2Li + O_2_ → Li_2_O_2_ (*E*_0_ = 2.96 V vs. Li/Li^+^)

Solid‐state LOBs alleviate issues such as Li corrosion and electrolyte decomposition, but their solid electrolytes typically suffer from low ionic conductivity. Consequently, their discharge capacities and cycle life are generally inferior to those of liquid‐electrolyte systems [42]. For these reasons, current research efforts predominantly focus on non‐aqueous LOBs employing proton‐inert organic electrolytes, which exhibit superior reversibility and cycling stability. The present work is also conducted within the framework of this non‐aqueous LOB system.

In non‐aqueous LOBs, the cell operates as an open system. During discharge, metallic Li at the anode is oxidized to Li^+^, which migrates through the electrolyte toward the O_2_ cathode. Simultaneously, molecular O_2_ from the surrounding environment diffuses into the porous cathode and, under the catalytic action of the electrode materials, is reduced to O_2_^−^. At the gas‐liquid‐solid three‐phase interface, comprising O_2_, the electrolyte, and the cathode surface, the reduced O species subsequently react with Li^+^ to form the discharge products. The overall reaction pathway is generally expressed as: O_2_ + e^−^ → O_2_^−^ and Li^+^ + O_2_ → LiO_2_, indicating that the ORR occurs first at the cathode side. Because LiO_2_ is thermodynamically unstable, the solid discharge product Li_2_O_2_ typically forms through two concurrent pathways: a disproportionation reaction and an electrochemical reduction process [33]. Both routes ultimately yield the same overall transformation: 2LiO_2_ → Li_2_O_2_ + O_2_ and LiO_2_ + Li^+^ + e^−^ → Li_2_O_2_.

During charging, the discharge product Li_2_O_2_ is decomposed under the applied potential and the catalytic action of the electrode materials, generating Li^+^ while releasing O_2_, as summarized in Table 1 [43]. However, because Li_2_O_2_ is an insoluble and electronically insulating solid in non‐aqueous electrolytes, its continuous accumulation progressively blocks the pores of the O_2_ electrode and covers the catalytic active sites, resulting in rapid deterioration of electrochemical performance. In addition, highly reactive intermediates formed during discharge, such as LiO_2_ and Li_2_O_2_, can attack the electrolyte and react with carbon materials, leading to severe parasitic reactions. Consequently, the practical application of non‐aqueous LOBs remains challenging. The key issues can be summarized as follows:

- Large charge/discharge overpotentials and low energy efficiency [20]. Although the equilibrium potential of a LOB is 2.96 V, the practical charge/discharge voltage gap is typically very large, resulting in energy efficiency far below the ∼90% required for commercial use. This problem primarily originates from the insulating nature of Li_2_O_2_, which is neither soluble nor conductive in non‐aqueous electrolytes. As discharge proceeds, Li_2_O_2_ accumulates within the cathode pores, blocking transport pathways and passivating catalytic sites, thereby slowing reaction kinetics. The intrinsic room‐temperature conductivity of Li_2_O_2_ is only 10^−9^‐10^−12^ S cm^−1^, severely limiting charge transport and causing the operating potentials to deviate significantly from the equilibrium value. Therefore, developing porous, highly active bifunctional catalysts or introducing suitable soluble RMs is essential for improving energy efficiency.
- Poor rate capability. The LOB relies on gas‐solid interfacial reactions involving a two‐phase ORR/OER process, which is intrinsically slower than gas‐liquid or homogeneous reactions. Under high current densities, Li_2_O_2_ forms rapidly, leading to rapid catalyst passivation and an increase in electrode resistance, which accelerates performance decay and prevents high‐rate operation [44]. To overcome this limitation, catalysts that promote the solution‐mediated formation of Li_2_O_2_ or guide its controlled solid‐state growth are needed to avoid sluggish two‐phase conversion. Furthermore, optimizing the porosity and electronic conductivity of the O_2_ electrode can significantly enhance rate performance.
- Short cycling life. Reactive ORR intermediates attack carbon cathodes and non‐aqueous electrolytes, causing structural degradation and electrolyte decomposition. This leads to the formation of high‐voltage parasitic products such as Li_2_CO_3_, which severely shorten cycling life [45]. Additionally, incomplete decomposition of Li_2_O_2_ due to limited catalytic activity results in its gradual accumulation, blocking active sites and pores. Over cycling, the charging voltage progressively increases, and excessively high charging potentials further destabilize carbon cathodes and electrolytes, triggering a detrimental self‐accelerating degradation loop. The development of more electrochemically stable electrolytes or non‐carbon cathode catalysts is therefore crucial for achieving long‐life LOBs.
- Safety concerns associated with the Li metal anode. As an open system, the LOB exposes highly reactive metallic Li to external gases and impurities, making parasitic reactions unavoidable. Moreover, uncontrolled Li deposition leads to dendrite formation, which can pierce the separator and cause short circuits or even fires. Repeated Li plating/stripping induces substantial volume fluctuations, destabilizing the solid electrolyte interphase and resulting in continuous consumption of both Li and electrolyte [46, 47], as well as increased polarization. Thus, implementing effective strategies to protect the Li metal anode is essential for improving the safety and stability of LOBs.

## 3 Recent Advances in Cathode Catalyst Materials for LOBs

The surface of the O_2_ cathode, where the redox reactions occur, plays a decisive role in governing the overall performance of LOBs (Figure 1e) [33]. According to the reaction mechanisms and the physicochemical characteristics of the discharge products, a high degree of porosity and abundant catalytically active surface sites are widely recognized as essential attributes for effective cathode materials.

Guided by these design principles, significant progress has been made in recent years toward developing high‐efficiency cathode catalysts that exhibit improved reaction kinetics and reversibility for LOBs [48]. Cathode catalysts reported to date can be broadly categorized into four major classes: carbon‐based materials, noble‐metal‐based catalysts, TM‐based oxides and compounds, and soluble catalyst systems. Each category offers distinct structural and catalytic advantages, while also presenting unique challenges that continue to shape ongoing research efforts.

Despite the attractive properties of non‐TM catalyst systems, including carbon materials and soluble catalysts, their intrinsic limitations remain significant in non‐aqueous LOBs. Carbon‐based materials primarily act as conductive frameworks rather than intrinsically active catalytic centers, and their catalytic activity strongly depends on defect sites and heteroatom functionalization. More importantly, carbon surfaces are susceptible to parasitic reactions with reactive oxygen species and Li_2_O_2_, particularly at elevated charging potentials, resulting in irreversible products such as Li_2_CO_3_ and severe electrode degradation [49]. Soluble RMs, although capable of regulating Li_2_O_2_ formation/decomposition through solution‐mediated pathways, often suffer from shuttle effects, mediator degradation, and complex side reactions with electrolytes and Li metal. In contrast, TM‐based catalysts provide tunable electronic structures through variable d‐orbital occupations, allowing stronger control over oxygen adsorption/desorption and reaction intermediates. Therefore, the development of TM‐based catalysts with optimized electronic structures remains a key strategy for achieving efficient bifunctional ORR/OER activity in LOB systems.

### 3.1 Carbon‐Based Catalyst Materials

**FIGURE 2:**
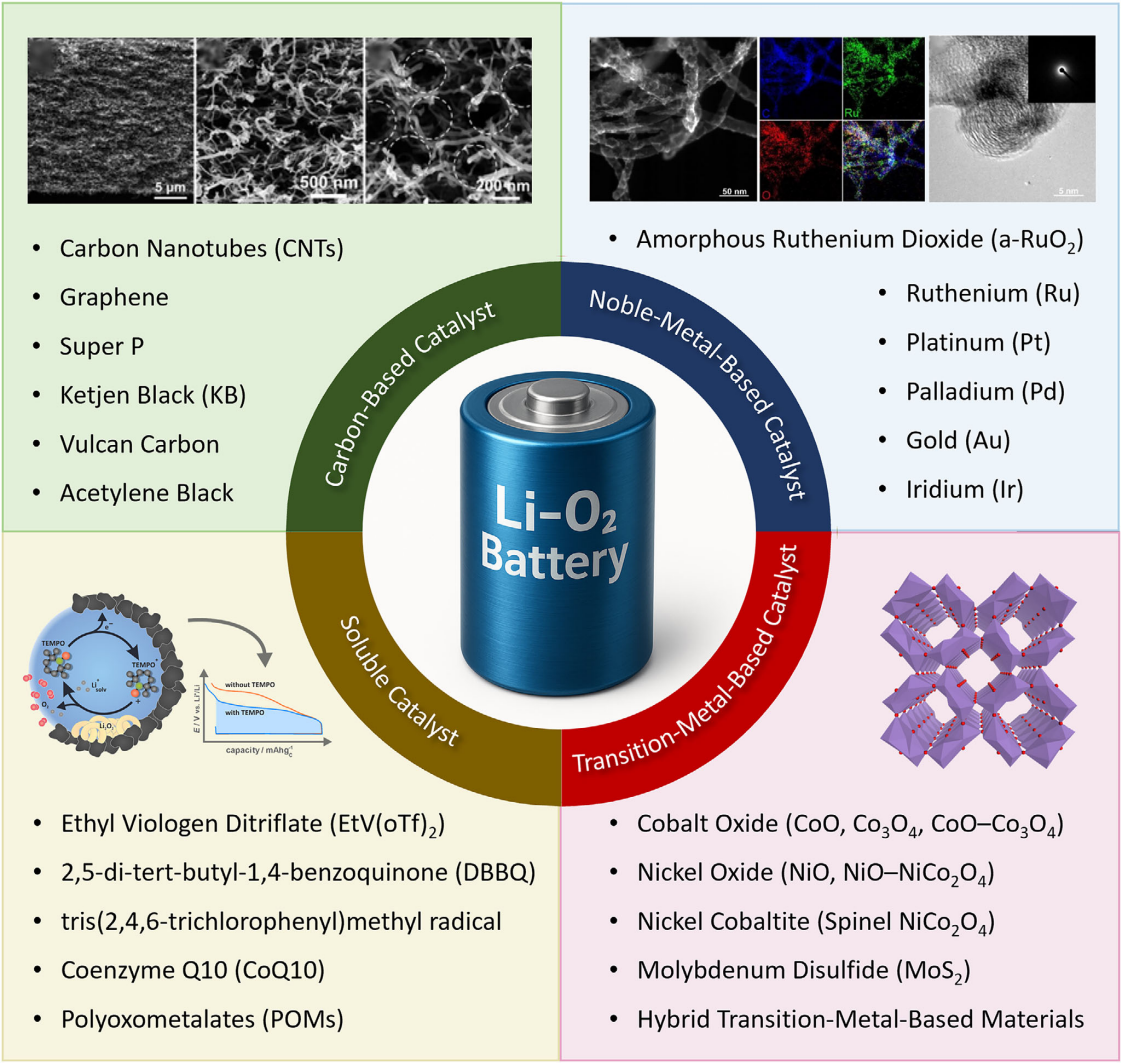
Various types of cathode catalyst materials for LOBs. Reproduced with permission [67]. Copyright 2013, Royal Society of Chemistry. Reproduced with permission [68]. Copyright 2021, Elsevier. Reproduced with permission [69]. Copyright 2014, American Chemical Society.

Carbon materials have been extensively employed in LOBs owing to their high electrical conductivity, low cost [50, 51], and the ease with which 3D porous architectures can be constructed (Figure 2). In addition to commercial conductive carbons such as Super P [52], Ketjen Black (KB) [53], Vulcan carbon [54], and acetylene black [55], a variety of functional carbon materials with unique microstructures, including 1D carbon nanotubes (CNTs), 2D graphene [56, 57], and 3D porous carbon nanostructures [58, 59], have demonstrated promising potentials [60].

Vertically aligned CNT arrays fabricated through chemical vapor deposition possess a highly ordered structure that promotes efficient O_2_ diffusion within the cathode while simultaneously exposing abundant active sites for redox reactions [61]. Such an architecture facilitates mass transport and interfacial charge transfer processes, leading to improved reaction kinetics and enhanced cycling stability in LOBs. In addition, porous CNT electrodes prepared at different synthesis temperatures exhibited significant differences in electrochemical behavior, with optimized structures delivering remarkable discharge capacities [62]. Variations in graphitization degree and the abundance of O‐containing functional groups on the carbon surface were found to strongly influence the electrochemical response of the electrodes by affecting conductivity and surface reaction characteristics.

N‐doped 3D graphene aerogels synthesized through a hydrothermal strategy feature a hierarchical macroporous–mesoporous framework that effectively facilitates O_2_ diffusion, promotes complete electrolyte infiltration, and accommodates the deposition of solid discharge products within the cathode structure [63]. The interconnected porous network alleviates mass transport limitations and provides sufficient reaction space, enabling a high specific capacity of approximately 7300 mAh g^−1^ while maintaining around 4000 mAh g^−1^ even at a current density of 200 mA g^−1^. Structural regulation through templating strategies has also been employed to optimize pore architectures. Microporous graphene papers and porous CNT membranes prepared using polystyrene colloidal spheres as sacrificial templates exhibited enlarged pore channels that allowed the reversible formation and decomposition of Li_2_O_2_ within the cathode framework [64]. In parallel with pore‐structure engineering, heteroatom doping, such as N, P, B, and S incorporation, has been explored as an effective approach for modifying the electronic structure and surface chemistry of carbon materials [65].

However, carbon materials are prone to instability during electrochemical cycling, which negatively affects long‐term durability. Bruce et al. employed differential electrochemical mass spectrometry and infrared spectroscopy to analyze cycled electrodes and gaseous products [66]. They reported that carbon materials remain stable below 3.5 V. However, above this threshold, carbon not only reacts with Li_2_O_2_ to form parasitic products such as Li_2_CO_3_ but may also undergo direct decomposition.

### 3.2 Noble‐Metal‐Based Catalyst Materials

Noble metals possess partially filled d orbitals, high electrochemical stability, and moderate adsorption strengths toward reaction intermediates, enabling significant enhancement of the reaction kinetics of the OER/ORR. Noble metals and their compounds, including Pt, Pd, Au, Ru, and Ir, have demonstrated exceptional catalytic performance in LOBs (Figure 2) [70, 71, 72, 73, 74, 75].

The use of dimethyl sulfoxide electrolytes combined with porous gold cathodes has demonstrated that optimizing both the reaction medium and cathode architecture can effectively improve the electrochemical behavior of LOBs [76]. The porous metallic framework facilitates reactant transport and interfacial reactions, allowing the system to maintain 95% capacity retention and stable cycling over 100 cycles even under high current densities. Further enhancement of cathode structures through integrated electrode design has been achieved by constructing interconnected porous Pd networks [77]. The continuous porous framework improves electron transport and provides accessible reaction pathways, leading to a cycling life exceeding 200 cycles under a fixed cutoff capacity. Hierarchical catalyst configurations integrating conductive frameworks and active catalytic sites have also been explored to regulate reaction kinetics and discharge‐product deposition behavior. N‐doped hollow carbon spheres loaded with Ir nanoparticles employed mesoporous ultrathin shells to accelerate mass transport, while the hollow interior created sufficient space for accommodating solid Li_2_O_2_ deposition [78]. Uniformly distributed Ir nanoparticles provided highly exposed catalytic surfaces that promoted electrochemical reactions and reduced charging overpotential. Amorphous RuO_2_ catalysts prepared through hydrothermal approaches further highlight the role of structural disorder in catalytic regulation. The presence of abundant defects, vacancies, and intrinsically disordered domains strengthened the adsorption of O_2_^−^ and LiO_2_ intermediates, enabling modulation of the particle size, morphology, crystallinity, and distribution of Li_2_O_2_ [68].

Despite their outstanding catalytic properties, the scarcity and high cost of noble metals hinder their large‐scale deployment. Therefore, strategies that maximize catalytic utilization and minimize the required loading of rare metals such as Pt and Ir will ultimately determine the industrial feasibility of noble‐metal‐based catalysts in LOBs.

### 3.3 Soluble Catalysts (RMs)

In non‐aqueous LOBs, the discharge products are insoluble and electronically insulating in organic electrolytes, often leading to unstable interfacial contact and severe blockage of the cathode pores. As a result, systems employing solely solid‐state catalysts still suffer from sluggish reaction kinetics and poor rate capability. By contrast, soluble catalysts, also referred to as RMs, exhibit favorable mobility within the electrolyte, enabling homogeneous dispersion and intimate contact with the entire surface of the discharge products. This unique characteristic has attracted considerable attention in recent years (Figure 2) [79].

According to their functional roles in LOBs, RMs can be broadly categorized into two types: ORR‐type RMs and OER‐type RMs. The following sections will discuss each category in detail, highlighting their catalytic mechanisms, representative molecular systems, and corresponding electrochemical benefits.

Although RMs are commonly categorized according to their dominant function in either ORR or OER processes, the distinction originates fundamentally from differences in their electronic structures and redox characteristics at the molecular level. ORR‐type RMs generally possess relatively low reduction potentials and electron‐rich states that facilitate electron donation to O_2_ molecules or LiO_2_ intermediates, thereby accelerating oxygen activation and promoting solution‐mediated Li_2_O_2_ formation [80]. By contrast, OER‐type RMs typically exhibit higher oxidation potentials and more stable oxidized states, enabling them to chemically oxidize Li_2_O_2_ during charging. From an electronic‐structure perspective, these behaviors can be interpreted in terms of differences in the density of states (DOS) near the Fermi level. ORR‐type mediators generally exhibit a higher DOS around occupied states that promotes electron transfer toward oxygen species, whereas OER‐type mediators favor accessible unoccupied states capable of accepting electrons from Li_2_O_2_ oxidation processes. Therefore, the energy‐level alignment and DOS distribution of redox mediators play critical roles in determining their preferred catalytic functionality.

#### 3.3.1 ORR‐Type RMs

ORR‐type RMs are typically capable of altering the formation pathway of Li_2_O_2_ during discharge. Their ability to enhance the ORR in LOBs generally stems from two primary mechanisms. First, through reversible changes in their oxidation states, RMs can accelerate electron transfer between the cathode surface and molecular O_2_ during the electrochemical reaction. Second, RMs can react with the LiO_2_ intermediate to form abundant RM‐LiO_2_ adducts, thereby inducing the solution‐mediated formation of Li_2_O_2_. This process effectively prevents catalyst passivation at the cathode surface and significantly increases the discharge capacity [81].

Lacey et al. first introduced ethyl viologen ditriflate (EtV(oTf)_2_) as a RM to improve ORR performance in LOBs [82]. During discharge, EtV(oTf)_2_ facilitates the reduction of O_2_ and promotes the disproportionation‐driven formation of Li_2_O_2_. However, because its redox potential (∼2.4 V vs. Li/Li^+^) is relatively low, a substantial portion of O_2_ still undergoes direct electrochemical reduction on the cathode surface, leaving the electrode passivation issue unresolved.

2,5‐di‐tert‐butyl‐1,4‐benzoquinone (DBBQ) was later introduced as a RM in low‐polarity and weakly solvating electrolytes [83]. Compared with earlier systems, DBBQ more effectively redirects the ORR pathway toward solution‐mediated processes, thereby suppressing direct Li_2_O_2_ accumulation on the cathode surface. Experimental results demonstrated substantially reduced discharge overpotentials together with an 80–100‐fold increase in discharge capacity. More recent investigations have further indicated that trace amounts of water in the electrolyte can strengthen the solution‐mediated pathway, accompanied by a morphological transition of Li_2_O_2_ from toroidal particles to plate‐like structures, which contributes to lower overpotentials (Figure 2). However, excess water reacts with Li metal to form parasitic products and introduces severe safety concerns [84, 85].

Beyond these examples, several other species, including tris(2,4,6‐trichlorophenyl)methyl radicals [86], coenzyme Q10 [87], and inorganic polyoxometalates [88], have also been identified as effective ORR‐type RMs capable of enhancing the electrochemical performance of LOBs.

#### 3.3.2 OER‐Type RMs

OER‐type RMs typically promote the decomposition of Li_2_O_2_ through a chemical oxidation pathway. In this process, the mediator is first electrochemically oxidized at the cathode surface to form RM^+^, which subsequently oxidizes Li_2_O_2_ chemically, generating Li^+^ and releasing O_2_ [89]. A wide variety of OER‐type RMs have been reported, falling broadly into three categories: organic molecules, organometallic salts, and inorganic compounds.

In 2013, Bruce and co‐workers first introduced tetrathiafulvalene [90], an organic redox‐active molecule, as an OER‐type RM for LOBs, achieving a substantial improvement in cycling stability. Bergner et al. demonstrated that 2,2,6,6‐tetramethylpiperidinyl‐1‐oxyl possesses high electrochemical stability [69], fast diffusion kinetics, and an appropriate redox potential, enabling it to markedly lower the charging overpotential to 3.5–3.7 V. Whan Shin and colleagues functionalized phenothiazine with ammonium‐based ionic liquid groups [91], creating a mediator capable of avoiding undesired reactions with Li metal while significantly improving cycling performance.

In addition, halide‐based RMs containing ions such as I^−^ and Br^−^, including LiI and LiBr, have been shown to participate in Li_2_O_2_ decomposition through reversible changes in the oxidation state of the halide species [92, 93]. This catalytic mechanism effectively accelerates the charging reaction and substantially reduces the charging overpotential, providing a promising approach for enhancing the reversibility of LOBs.

Although the introduction of soluble RMs has opened new avenues for improving the electrochemical performance of LOBs, their practical application remains constrained by several inherent challenges. In non‐aqueous electrolytes, soluble RMs inevitably undergo a “shuttle effect” [94], which complicates the reaction environment and introduces additional side processes. The involvement of RMs in multiple intertwined electrochemical and chemical pathways makes the overall reaction mechanism difficult to elucidate. Moreover, the shuttle effect can induce corrosion of the Li metal anode and lead to the continuous consumption of RM's own redox‐active sites [95], thereby diminishing their long‐term effectiveness.

For these reasons, further investigation is required to understand, control, and ultimately mitigate the limitations associated with soluble RMs. Continued research into their reaction mechanisms, stability, and interactions with electrode/electrolyte components will be essential for enabling their practical deployment in high‐performance LOBs.

### 3.4 TM‐Based Catalyst Materials

Driven by cost considerations, researchers have increasingly turned to TMs and their oxides, carbides, and nitrides as alternative catalyst materials. Reported TM‐based catalysts primarily include Mn‐based materials, Co‐based materials, spinel‐type oxides, and perovskite structures. Owing to their abundant unstable electronic orbitals and unpaired electrons, these materials typically exhibit intrinsic catalytic activity toward the reactions involved in LOBs [96, 97, 98, 99].

Beyond their compositional diversity, the catalytic performance of TM‐based materials is fundamentally governed by their electronic configurations at the atomic scale. In bifunctional catalysts for LOBs, the adsorption and conversion of oxygen intermediates (O^2−^, LiO_2_, and Li_2_O_2_) strongly depend on the occupancy and energy distribution of TM d orbitals [100]. Excessively weak adsorption leads to insufficient activation of O_2_ during ORR, whereas overly strong adsorption hampers the decomposition of Li_2_O_2_ during OER. Therefore, highly efficient bifunctional catalysts generally require a moderate electronic configuration capable of balancing adsorption and desorption energies. Previous studies suggest that optimized e_g_ orbital occupancy close to unity and a suitable d‐band center position can facilitate intermediate binding while minimizing reaction barriers. Such electronic‐structure regulation provides an important design principle for developing highly active bifunctional catalysts in LOB systems.

The construction of rational catalyst architectures has been widely explored to improve the electrochemical performance of LOBs by increasing active‐site exposure and facilitating reactant transport. In this context, 3D hollow α‐MnO_2_ frameworks with porous shells were synthesized through a sacrificial‐template strategy and employed as cathode catalysts [96, 101]. The hollow and porous structural characteristics are beneficial for electrolyte penetration and oxygen diffusion while providing sufficient space for discharge‐product accommodation. As a result, the corresponding cells delivered a high specific capacity of 8583 mAh g^−1^ at 100 mA g^−1^ and retained 6311 mAh g^−1^ at 300 mA g^−1^, together with stable cycling for 170 cycles at 200 mA g^−1^. Morphology engineering has also been shown to influence catalytic activity and transport behavior significantly. Sea‐urchin‐like NiO‐NiCo_2_O_4_ microspheres prepared through a hydrothermal route exhibited a hierarchical structure capable of improving ion and oxygen accessibility [102]. The assembled battery achieved a discharge capacity of 9231 mAh g^−1^ at 100 mA g^−1^ and maintained 3711 mAh g^−1^ at 500 mA g^−1^, indicating favorable rate capability. Under a current density of 100 mA g^−1^ with a limited capacity of 600 mAh g^−1^, stable cycling was maintained for 80 cycles.

Beyond structural optimization of metal oxides, integrating catalytic materials with conductive carbon frameworks provides an effective approach for improving charge transport and electrode stability. A binder‐free and self‐supporting air electrode based on 1T‐MoS_2_ and functionalized CNTs was fabricated via an in‐situ liquid‐phase redox intercalation and exfoliation process [103]. The conductive network formed by CNTs, together with the catalytic contribution of 1T‐MoS_2_, enabled stable battery operation for 100 cycles at 200 mA g^−1^ with a cutoff capacity of 500 mAh g^−1^. Further enhancement in catalytic performance has been achieved through the synergistic combination of active nanoparticles and heteroatom‐doped carbon substrates. Uniform anchoring of CoO‐Co_3_O_4_ nanoparticles onto N‐doped hollow carbon spheres generated a catalyst structure with abundant exposed active sites and a large accessible surface area [104]. Benefiting from these structural characteristics, the battery delivered an ultrahigh discharge capacity of 24265 mAh g^−1^ at 300 mA g^−1^, while cells employing the N‐HC@CoO‐Co_3_O_4_ cathode maintained stable cycling for more than 112 cycles at a cutoff capacity of 500 mAh g^−1^.

### 3.5 Critical Considerations in Performance Evaluation

Although numerous studies have reported significant improvements in discharge capacity, cycling life, and rate performance of LOBs, direct comparison among different catalyst systems remains challenging because electrochemical performance is highly dependent on testing conditions [105, 106]. Parameters including electrolyte composition, O_2_ purity, current density, electrode loading, cathode architecture, and capacity limitations can strongly influence reported results.

In many cases, exceptionally high discharge capacities are obtained under very low current densities or low cathode mass loadings, which may not represent realistic operating conditions. Since capacity values are frequently normalized to catalyst or electrode mass, low loading levels can artificially produce very high specific capacities while contributing little to practical energy density at the device level. Furthermore, long cycling stability is often evaluated using capacity‐limited cycling protocols [107]. Under such conditions, the discharge process is intentionally terminated before substantial accumulation of Li_2_O_2_ occurs, thereby reducing electrode passivation and mitigating degradation processes. While this approach provides useful information regarding catalyst reversibility, it may significantly overestimate long‐term cycling stability under practical operating conditions.

Therefore, it is important to distinguish between metrics suitable for academic benchmarking and those relevant to practical implementation [108]. Academic studies often prioritize understanding reaction mechanisms and evaluating intrinsic catalytic activity under controlled conditions. By contrast, practical LOB systems require simultaneous consideration of factors including areal capacity, electrode loading, energy efficiency, long‐term stability, and realistic operating environments. Consequently, future studies would benefit from more standardized testing protocols and reporting criteria, enabling fair comparison among catalyst systems and facilitating translation from laboratory‐scale demonstrations toward practical LOB devices.

## 4 MnO_x_‐Based Catalysts and Their Applications in LOBs

Mn element, a first‐row TM with five unpaired electrons, possesses a distinct electronic configuration [109, 110], which endows it with high redox activity. This enables Mn to form a wide range of MnO_x_, including MnO, MnO_2_, Mn_2_O_3_, Mn_3_O_4_, Mn_2_O_5_, MnO_3_, and Mn_2_O_7_. In addition, the diverse connection modes of the fundamental [MnO_6_] octahedral units give rise to a rich variety of MnO_2_ crystal structures [111, 112], commonly classified into six major polymorphs: α, β, δ, λ, γ, and ε [99].

Mn is also abundant in the Earth's crust, ranking 12^th^ among crustal elements with an approximate content of 0.1%. Compared with many other TM oxides, MnO_x_ are considerably more cost‐effective. The wide range of accessible oxidation states and polymorphic structures provides MnO_x_‐based materials with exceptional synthetic versatility and tunable physicochemical properties, making them highly attractive as catalysts for LOBs.

Owing to the favorable properties of MnO_x_, considerable progress has been achieved in recent years in the development of MnO_x_‐based catalysts for LOBs. Table 2 summarizes the representative electrochemical performance of MnO_x_‐based LOB cathodes reported over the past three years (capacities normalized to catalyst mass).

**TABLE 2:** Statistics of the representative performance of MnO_x_‐based cathodes over the past three years.

Cathode Material	Year	Current Density (mA g^−1^)	Capacity of Catalyst Materials (mAh g^−1^)	Retention (cycles)	Refs.
Ru‐doped β‐MnO_2_	2025	1000	9300	54.2%/720 h	[113]
In_2_S_3_/MnO_2_/BiOCl	2025	100	19330	∼100/1500 h	[114]
Carbon/RuO_2_/MnO_2_	2025	0.1 mA cm^−2^	5.24 mAh cm^−2^	–/730 h	[115]
Ru@MnCo_2_O_4_/Carbon	2024	200	27810	∼100%/106	[116]
Mn_3_O_4_@CNT	2024	200	16895	∼100%/97	[117]
MnCo_1.75_Cu_0.25_O_4_	2024	175	11272	–/1250 h	[118]
MnTe/MnO	2024	200	11930	–/350	[119]
0.5La_0.6_Sr_0.4_MnO_3_‐0.5LaMn_0.6_Co_0.4_O_3_	2024	300	14499	∼100%/258	[120]
Ru@La(Mn_0.2_Co_0.2_Fe_0.2_Ni_0.2_Cr_0.2_)O_3_	2024	50	12275	∼100%/345	[121]
(La_0.8_Sr_0.2_)(Mn_0.2_Fe_0.2_Cr_0.2_Co_0.2_Ni_0.2_)O_3_	2024	100	17078	–/435	[122]
Co‐doped MnCr_2_O_4_	2023	200	13600	∼100%/150	[123]
Cr‐doped Mn_3_O_4_	2023	100	22288	∼100%/74	[124]
Mn_2_O_3_	2023	500	21070	∼100%/180	[125]
FeMnO_3_@ZrO_2_	2023	50	11800	∼100%/100	[126]
ε‐MnO_2_	2023	200	1982	48.1%/100	[30]
MnO_2_‐Co_3_O_4_@CC	2023	100	8115	–/125	[127]
Mn_2_O_3_@CeO_2_	2023	1000	12545	–/300	[128]
Co_2.85_Mn_0.15_O_4_	2023	1000	5598	∼100%/100	[129]
Ru@MnO_2_	2023	0.05 mA cm^−2^	7.74 mAh cm^−2^	∼100%/1800 h	[130]

In the following sections, we discuss the application of MnO_x_‐based catalysts in LOBs from three perspectives: monometallic MnO_x_, bimetallic MnO_x_, and multimetallic MnO_x_. Each category offers distinct advantages in catalytic activity, structural engineering, and reaction‐pathway regulation, enabling tailored optimization of LOB performance.

### 4.1 Monometallic MnO_x_ as Catalysts for LOBs

**FIGURE 3:**
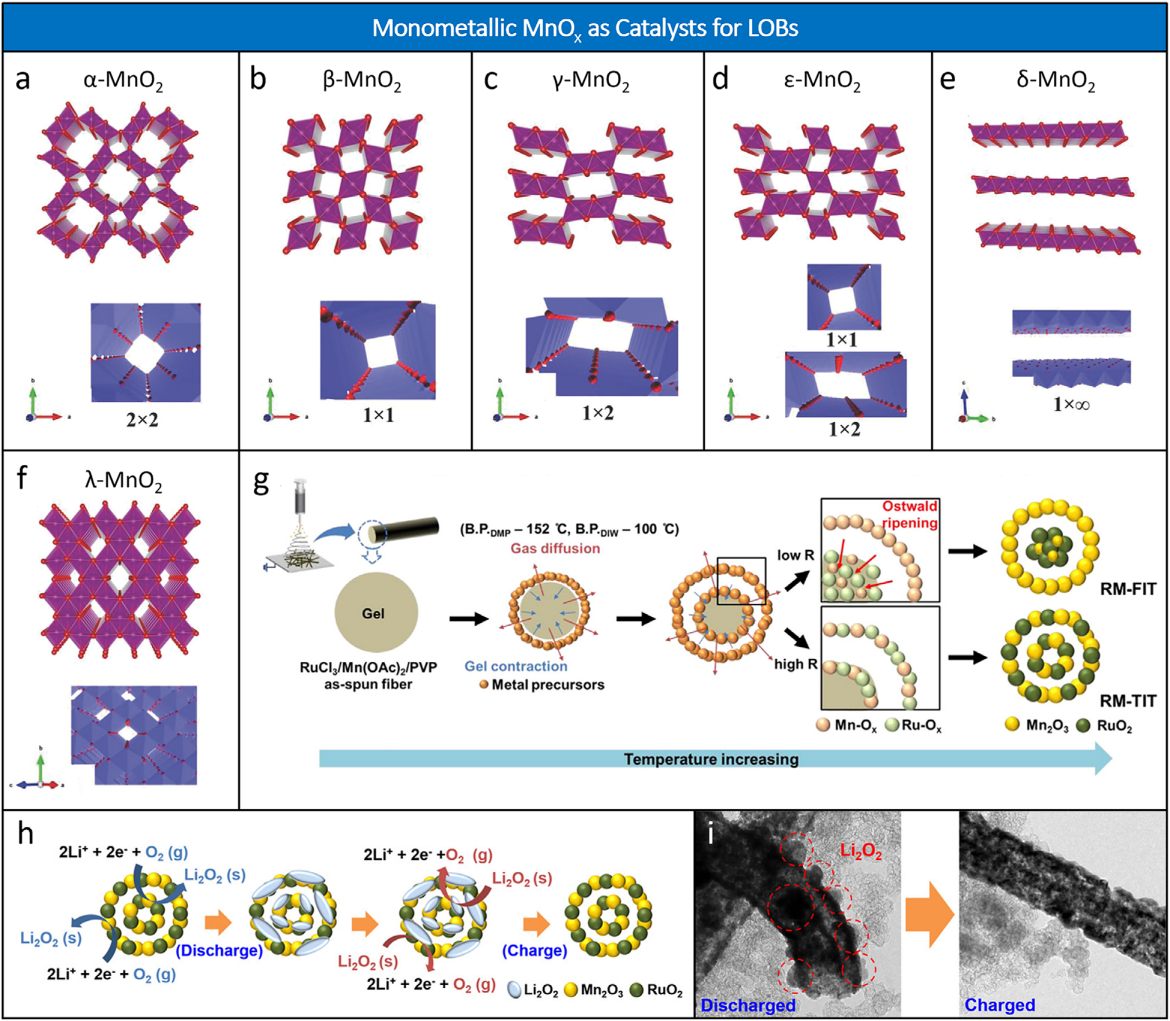
Crystal structures and catalytic behavior of representative MnO_x_ catalysts for LOBs. (a‐f) Schematic diagrams for crystal structures of MnO_2_: α‐MnO_2_ (a), β‐MnO_2_ (b), γ‐MnO_2_ (c), ε‐MnO_2_ (d), δ‐MnO_2_ (e), and λ‐MnO_2_ (f). Reproduced with permission [131]. Copyright 2018, Wiley. (g) Schematic illustration of the fabrication process of RuO_2_/Mn_2_O_3_ tubular catalysts. (h‐i) Schematic representation of the reversible formation and decomposition processes of Li_2_O_2_ during discharge and charge, respectively. Reproduced with permission [132]. Copyright 2016, American Chemical Society.

Since the Bruce group first demonstrated in 2006 that MnO_2_ can serve as an effective catalyst in LOBs, thereby achieving promising electrochemical performance, extensive research efforts have been devoted to exploring MnO_x_ materials [133, 134]. Among the various MnO_x_, MnO_2_ exhibits the richest polymorphism and can be categorized into three major structural families: 1D tunnel structures (α, β, γ, and ε phases), 2D layered structures (δ phase), and 3D framework structures (spinel‐type λ phase) (Figure 3a–f) [131].

α‐MnO_2_ contains a 2 × 2 tunnel with a diameter of approximately 0.46 nm, providing ample space for the reversible formation and decomposition of Li_x_O_y_ during cycling, as well as facilitating rapid diffusion of O_2_, Li_x_O_y_ intermediates, and Li^+^ [135]. The catalytic activity of MnO_2_ polymorphs is generally ranked as α‐MnO_2_ > amorphous MnO_2_ > β‐MnO_2_ > δ‐MnO_2_ [136]. These findings highlight that the crystal structure of MnO_2_ critically influences both its catalytic activity and the reaction pathways of the ORR/OER processes in LOBs.

In addition to crystal structure, the nanoscale dimensions and morphological features of MnO_x_ also play a decisive role in determining their electrocatalytic behavior. For example, the Bruce group reported that nanorod‐shaped α‐MnO_2_ exhibits an initial discharge capacity of approximately 3000 mAh g^−1^ [137]. significantly outperforming bulk α‐MnO_2_, while also offering improved cycling stability. Suib and co‐workers compared the catalytic activities of α‐MnO_2_ with different morphologies in both aqueous and non‐aqueous electrolytes [138]. Among all the samples studied, solvent‐free synthesized nanorods displayed the highest ORR activity, attributed to their lower average Mn oxidation state, smaller crystallite size, larger surface area, and higher pore volume, as confirmed by limiting and exchange current densities.

To improve catalytic activity and mass transport behavior simultaneously, a 3D δ‐MnO_2_ aerogel was constructed through a combined wet‐chemical synthesis and liquid N_2_ freeze‐drying process [139]. The interconnected porous framework and high specific surface area provided abundant exposed catalytic sites while promoting effective diffusion of oxygen and electrolyte throughout the electrode structure. These structural features contributed to enhanced reaction kinetics and resulted in excellent electrocatalytic performance during LOB operation.

Although MnO_2_ has repeatedly been demonstrated as an efficient catalyst for LOBs, theoretical calculations suggest that it may react with discharge products to form MnO_2_‐Li_x_O_y_ intermediates, which could lead to structural instability during cycling [135]. Consequently, MnO_x_ with lower average oxidation states, such as MnO, Mn_2_O_3_, and Mn_3_O_4_, have attracted increasing attention due to their greater structural stability and promising catalytic properties.

The integration of Mn‐based oxides with conductive carbon frameworks has been widely explored to simultaneously improve catalytic activity and charge transport properties. A MnO@CNT composite was synthesized through direct reaction between CNTs and KMnO_4_ followed by thermal reduction treatment [140]. The conductive CNT network provided efficient electron pathways, while the intrinsic stability and catalytic activity of MnO facilitated the electrochemical reactions involved in LOB operation. Under a current density of 0.4 mA cm^−2^ and a limited capacity of 1000 mAh g^−1^, the assembled cell maintained a discharge plateau above 2.4 V after 100 cycles. In addition to MnO, Mn_2_O_3_ and Mn_3_O_4_, as monometallic MnO_x_ with mixed valence states, have also attracted considerable interest. The coexistence of redox‐active metal ion pairs and lattice distortions associated with multivalent Mn species can generate additional active sites and alter the electronic structure, thereby enhancing electrocatalytic activity [141].

Further enhancement in catalytic performance can be achieved through the synergistic integration of multiple active components and tailored structural design. A RuO_2_/Mn_2_O_3_ bifunctional catalyst with a unique tubular architecture was fabricated using a simple electrospinning technique combined with controlled‐rate calcination (Figure 3g–i) [132]. The tubular morphology can facilitate reactant transport and provide greater accessibility to catalytic sites, while the combination of RuO_2_ and Mn_2_O_3_ enables cooperative catalytic effects during electrochemical reactions. Electrochemical analyses demonstrated that the RuO_2_/Mn_2_O_3_ catalyst exhibited higher OER activity than commercial Pt/C in alkaline media, and LOBs employing this catalyst maintained stable cycling over 100 cycles. The coupling of catalytic nanoparticles with conductive carbon frameworks has also been utilized to improve electron transport and catalyst utilization. Carbon nanofibers (CNFs) modified with Mn_3_O_4_ nanoparticles (Mn_3_O_4_/CNFs) were developed as catalysts for LOBs operating in a hybrid electrolyte [142]. Compared with electrodes prepared using Mn_3_O_4_ mixed with KB or pure KB, the Mn_3_O_4_/CNF‐KB composite significantly reduced charge/discharge overpotentials and enhanced cycling stability, which can be attributed to improved electrical conductivity and more effective dispersion of catalytic active sites.

### 4.2 Bimetallic MnO_x_ as Catalysts for LOBs

**FIGURE 4:**
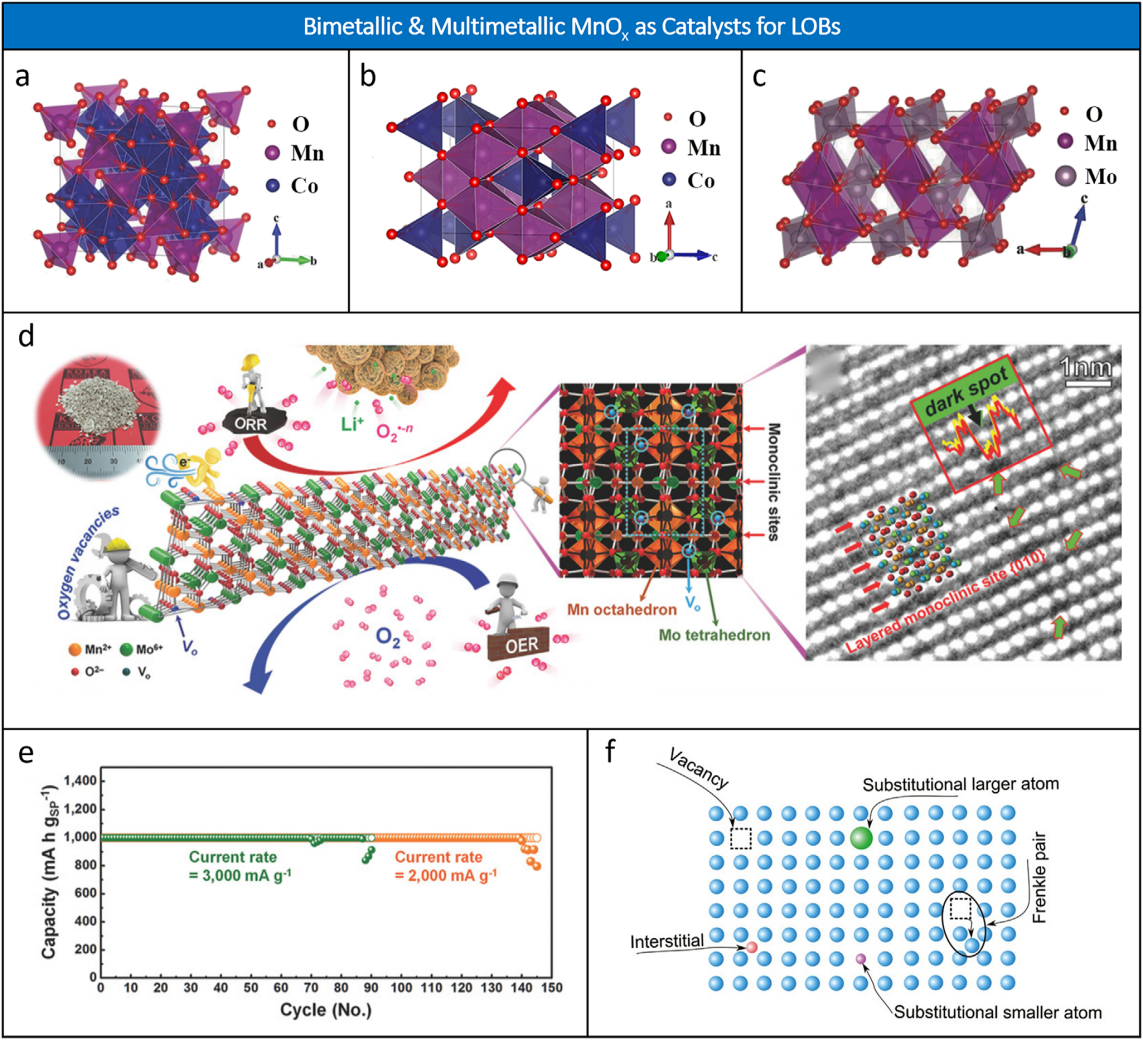
(a–c) Crystal structure of spinel‐type MnCo_2_O_4_ (a), inverse spinel‐type CoMn_2_O_4_ (b), and scheelite‐type MnMoO_4_ (c). (d) Catalytic mechanism for MnMoO_4_. (e) Cycle lifespan of MnMoO_4_ at different current density. Reproduced with permission [144]. Copyright 2017, Wiley. (f) Crystal defects in nonstoichiometric oxides of ABO_3_. Reproduced with permission [146]. Copyright 2015, American Chemical Society.

To harness the synergistic catalytic effects between different metal ions and further enhance the electronic conductivity of MnO_x_, extensive research has been devoted to bimetallic oxide systems such as AMn_2_O_4_, MnB_2_O_4_, ABO_4_, and AMnO_3_. MnB_2_O_4_ and AMn_2_O_4_ oxides adopt spinel and inverse‐spinel structures (Figure 4a,b), in which Mn^2+^ and Mn^3+^ occupy tetrahedral and octahedral sites within the crystal lattice. Here, B denotes trivalent metal ions such as Fe, Co, Al, and Cr, whereas A represents divalent TM ions including Fe and Co.

Structural regulation and compositional engineering of Mn‐based oxides have been extensively explored to optimize catalytic activity and electrochemical behavior in LOBs. Various spinel‐type materials with distinct morphologies, including nanorods, nanotubes, and tube‐in‐tube structures of NiMn_2_O_4_, ZnMn_2_O_4_, and CoMn_2_O_4_, were fabricated through electrospinning followed by controlled‐rate thermal treatment [143]. The structural diversity generated by this approach can influence mass transport pathways and active‐site accessibility. Among these materials, CoMn_2_O_4_ nanotubes exhibited superior electrochemical performance, enabling LOBs to deliver a discharge capacity of 5335 mAh g^−1^ at a current density of 100 mA g^−1^.

Tungsten‐bronze‐type ABO_4_ oxides have also attracted considerable attention because of their abundant O vacancies. When the concentration of A–O–B bridges becomes sufficiently high, the resulting anion vacancies can introduce intrinsic n‐type conductivity into ABO_4_ MnO_x_ systems. MnMoO_4_ nanowires synthesized via co‐precipitation followed by a solid‐state reaction demonstrated that O vacancies generated through dual‐metal‐ion coordination play an important role in regulating electrochemical reversibility in LOBs (Figure 4c–e) [144]. Such defect structures can alter the electronic environment and facilitate charge transfer during electrochemical processes. The corresponding cells exhibited excellent rate capability and maintained stable cycling for 188 cycles at a limited capacity of 1000 mAh g^−1^. Even under increased current densities and larger capacity cutoffs, stable operation beyond 50 cycles was still maintained.

The AMnO_3_ family belongs to perovskite‐type MnO_x_ materials, in which A represents rare‐earth or alkaline‐earth metal ions. Within this structure, Mn atoms are octahedrally coordinated by six O atoms, forming a framework that can provide flexible electronic regulation and tunable catalytic behavior, with CaMnO_3_ being one of the most representative examples. Porous mesh‐like CaMnO_3_ nanoparticles prepared through a sol‐gel process generated a catalyst architecture with a large accessible surface area and interconnected transport pathways [145]. When employed in LOBs, the CaMnO_3_/C cathode effectively reduced reaction overpotentials and maintained stable cycling for more than 80 cycles.

### 4.3 Multimetallic MnO_x_ as Catalysts for LOBs

In perovskite‐type oxides (ABO_3_), partially substituting the A‐ or B‐site atoms with heteroatoms, while preserving the overall crystal structure, can significantly enhance electronic and ionic conductivity, introduce a higher concentration of defects, and improve lattice O mobility (Figure 4f) [146]. Such modifications offer an effective strategy to tune catalytic activity and stability for LOB applications.

The first to employ La_0.8_Sr_0.2_MnO_3_ as a catalyst for LOBs was by Débart et al. They investigated its influence on electrochemical performance [147]. Their results demonstrated that La_0.8_Sr_0.2_MnO_3_ serves as an efficient ORR catalyst, delivering a discharge potential of up to 2.6 V. Morphology engineering of perovskite‐type catalysts has been demonstrated to exert a significant influence on electrochemical performance through regulation of active‐site exposure and mass transport behavior. Porous La_0.75_Sr_0.25_MnO_3_ nanotubes (PNT‐LSM) prepared through electrospinning followed by annealing provided an interconnected porous framework capable of facilitating oxygen diffusion and electrolyte penetration [148]. Compared with pristine KB cathodes, the incorporation of PNT‐LSM increased the discharge potential by 30–50 mV and lowered the charging potential by 200 mV at a current density of 0.025 mA cm^−2^, while delivering a maximum discharge capacity of 11000 mAh g^−1^. When combined with Super P, the PNT‐LSM cathode further exhibited stable cycling for more than 124 cycles under a limited capacity of 1000 mAh g^−1^.

Beyond morphological optimization, compositional regulation of perovskite structures offers an additional strategy for tuning catalytic properties. Highly active oxidized La_x_Ca_1‐x_MnO_3_ nanoparticles with particle sizes of 20–35 nm were synthesized through an ionic‐liquid‐based approach [149]. Modifying the A‐site composition can induce simultaneous changes in both the geometric and electronic structures of the perovskite lattice, leading to shifts in the average Mn oxidation state and corresponding variations in catalytic activity.

### 4.4 Operando Characterization of MnO_x_‐Based Catalysts in LOBs

Recent advances in operando characterization techniques have significantly improved the understanding of dynamic processes occurring in LOB cathodes. Unlike ex situ measurements, operando approaches enable real‐time monitoring of reaction intermediates, discharge‐product evolution, and structural transformations during cycling. Techniques such as operando X‐ray diffraction (XRD), Raman spectroscopy, transmission electron microscopy (TEM), differential electrochemical mass spectrometry (DEMS), and X‐ray absorption spectroscopy (XAS) have been widely employed to probe Li_2_O_2_ formation/decomposition and catalyst evolution [150, 151, 152]. These techniques provide direct information regarding phase transitions, reaction pathways, gas evolution, and interfacial processes under practical operating conditions, thereby overcoming the limitations associated with post‐mortem characterization. In particular, they can distinguish between surface‐mediated and solution‐mediated reaction mechanisms and reveal the dynamic behavior of oxygen intermediates during discharge and charge processes.

For MnO_x_‐based catalysts, these techniques are particularly valuable because the catalytic activity strongly depends on the Mn oxidation state [125], crystal phase, and local coordination environment. For example, operando XAS can track variations in Mn valence states and local structural evolution during ORR/OER processes, while operando Raman and XRD can reveal the nucleation and decomposition behavior of Li_2_O_2_ and identify parasitic products. Moreover, operando DEMS enables direct detection of O_2_ consumption and evolution behavior, providing insights into reaction reversibility and side reactions associated with electrolyte decomposition. Advanced operando TEM can further visualize morphology evolution and the growth behavior of discharge products at the nanoscale [153]. Such real‐time observations provide direct evidence for catalyst–intermediate interactions and facilitate the establishment of structure–activity relationships for rational catalyst design. Furthermore, combining operando characterization with theoretical calculations, such as density functional theory, may enable deeper understanding of reaction energetics and dynamic catalytic mechanisms, thereby guiding the development of highly efficient MnO_x_‐based cathodes for practical LOB applications.

### 4.5 Comparison of MnO_x_ with Other Representative Cathode Catalysts

Although MnO_x_‐based catalysts have attracted considerable attention because of their low cost, abundant reserves, and diverse oxidation states, their performance should be evaluated in comparison with other representative catalyst systems employed in LOBs. Noble‐metal‐based catalysts, such as Pt‐, Ru‐, and Ir‐containing materials, generally exhibit superior intrinsic catalytic activity and lower overpotentials owing to their optimized electronic structures and favorable adsorption characteristics toward oxygen intermediates [154, 155, 156]. However, their practical implementation remains limited by high cost and scarcity.

Co‐based oxides and spinel structures have also demonstrated excellent bifunctional ORR/OER activity and often deliver higher electronic conductivity than MnO_x_ [157]. Similarly, Mn‐free perovskite catalysts can provide improved oxygen‐ion mobility and tunable electronic structures through A‐ and B‐site engineering. Nevertheless, these materials frequently involve more complex synthesis procedures and higher material costs. In contrast, MnO_x_‐based catalysts offer distinct advantages arising from their structural diversity, variable oxidation states, and ease of compositional modification [158, 159]. The availability of multiple polymorphs and defect‐engineering strategies enables flexible regulation of catalytic activity and reaction pathways. However, the relatively low intrinsic electronic conductivity of MnO_x_ and its potential structural evolution during cycling remain important limitations.

It should also be noted that the superior performance reported for some MnO_x_ systems may not solely originate from the intrinsic catalytic properties of MnO_x_ itself. Factors such as hierarchical electrode architectures, conductive carbon frameworks, heteroatom doping, and optimized testing conditions may contribute substantially to the observed improvements [160, 161, 162]. Therefore, meaningful evaluation of catalyst performance requires simultaneous consideration of catalyst composition, electrode design, and testing protocols. MnO_x_ should not necessarily be regarded as universally superior to competing catalyst systems, but rather as a highly promising and economically attractive platform with considerable potential for further optimization.

### 4.6 Electronic‐Structure Regulation and Mechanistic Considerations

The catalytic behavior of MnO_x_ in LOBs is fundamentally governed by its electronic structure, which determines the adsorption strength and conversion energetics of oxygen intermediates. Parameters including Mn oxidation state, oxygen vacancies, and metal–oxygen covalency play critical roles in regulating ORR/OER pathways and the reversible formation/decomposition of Li_2_O_2_.

Mn oxidation states strongly influence the occupation of Mn 3d orbitals and the electronic interaction with oxygen species [121]. Higher Mn valence states generally increase the covalent character of Mn─O bonds and modify the adsorption energies of O_2_^−^ and LiO_2_ intermediates. Moderate adsorption strength is desirable because excessively weak interactions may hinder oxygen activation, whereas overly strong adsorption can impede intermediate desorption and Li_2_O_2_ decomposition during charging. Oxygen vacancies are also widely recognized as active sites for enhancing catalytic activity [163]. Their introduction can alter local charge density and electronic distributions, facilitating oxygen adsorption and lowering reaction barriers. In several studies, oxygen‐deficient MnO_x_ has been associated with a transition from surface‐mediated Li_2_O_2_ growth toward solution‐mediated pathways because modified adsorption energies may reduce catalyst passivation and promote the growth of larger discharge products.

However, the mechanistic understanding remains incomplete. For example, whether oxygen vacancies directly induce solution‐mediated growth or instead alter local electrolyte environments remains under debate. Similarly, the relative contributions of disproportionation reactions and direct electrochemical reduction pathways in Li_2_O_2_ formation may vary depending on electrolyte properties, current density, and catalyst surfaces [164, 165]. During OER processes, the onset potential and kinetic behavior are strongly affected by the electronic interaction between Mn sites and oxygen‐containing intermediates. Previous studies suggest that stronger metal–oxygen covalency may facilitate charge transfer and reduce reaction barriers for Li_2_O_2_ oxidation [166]. Nevertheless, direct experimental evidence linking electronic descriptors to catalytic activity remains limited, and many current interpretations rely heavily on theoretical calculations and indirect spectroscopic observations.

Therefore, although relationships between catalyst electronic structures and catalytic performance have been widely proposed, many mechanistic conclusions remain qualitative or model‐dependent. Future studies integrating operando characterization with theoretical calculations are needed to establish quantitative structure–activity relationships and resolve existing mechanistic debates.

### 4.7 Chemical and Electrochemical Stability of MnO_x_ Catalysts

Although MnO_x_‐based catalysts have demonstrated promising catalytic activity and cycling performance in LOBs, their long‐term chemical and electrochemical stability under practical operating conditions remains a critical concern. In many reported systems, improved electrochemical performance does not necessarily imply intrinsic catalyst stability, because the catalyst itself may undergo structural evolution or participate in side reactions during repeated cycling.

One important degradation pathway involves Mn dissolution and crystal phase transformation during electrochemical operation [167]. Repeated ORR/OER processes can induce changes in local coordination environments and Mn oxidation states, leading to structural rearrangement of MnO_x_ phases. In particular, high‐valence Mn species may exhibit limited thermodynamic stability and become susceptible to reduction or structural collapse under prolonged cycling conditions [168]. Such phase evolution may alter active‐site distributions and gradually deteriorate catalytic activity.

Another possible degradation mechanism involves the formation of Mn–Li_x_O_y_ intermediate species during discharge and charge processes [169]. Previous theoretical studies have suggested that interactions between MnO_x_ surfaces and oxygen‐containing intermediates may lead to the formation of surface‐bound Mn–Li_x_O_y_ species [170]. While these intermediate structures may facilitate adsorption and activation of oxygen species, their excessive accumulation may induce irreversible surface reconstruction or block active catalytic sites. However, the precise role of Mn–Li_x_O_y_ intermediates remains incompletely understood because direct experimental observations remain limited.

In addition, MnO_x_ catalysts may participate in parasitic reactions involving electrolytes and carbon supports. Reactive oxygen species generated during ORR/OER processes [171], particularly superoxide intermediates and Li_2_O_2_‐related species, can attack both electrolytes and conductive carbon materials. Surface catalytic sites may further accelerate undesirable decomposition reactions under certain conditions [172], leading to the formation of irreversible products such as Li_2_CO_3_ and other side products. Such reactions can progressively block cathode pores and increase polarization during cycling.

Experimental studies employing operando XAS, Raman spectroscopy, and TEM have provided evidence of structural evolution and dynamic changes in catalyst surfaces during operation [116, 173], while theoretical calculations have suggested that variations in adsorption energies and local electronic structures can strongly affect catalyst stability [174]. Nevertheless, many degradation mechanisms remain difficult to distinguish because multiple processes, including electrolyte decomposition, catalyst reconstruction, and discharge‐product accumulation, often occur simultaneously.

Therefore, future studies should place greater emphasis on stability‐oriented catalyst design, including stabilization of Mn oxidation states, suppression of structural reconstruction, and mitigation of catalyst‐induced parasitic reactions. Combining advanced operando characterization techniques with theoretical modeling may further clarify degradation pathways and guide the development of durable MnO_x_‐based cathodes.

## 5 Modification Strategies for MnO_x_‐Based Cathode Catalysts

As discussed in the previous section, MnO_x_ have attracted extensive attention due to their low cost and intrinsically high catalytic activity. However, their catalytic efficiency, electronic conductivity, and overall electrochemical stability still fall short of meeting the stringent performance requirements for state‐of‐the‐art cathode catalysts in LOBs [175]. Accordingly, rationally tailoring the spatial architecture and surface physicochemical properties of MnO_x_‐based catalysts is of great significance for ensuring efficient mass and charge transport, reducing reaction overpotentials, and improving cycling stability during repeated charge/discharge processes.

To date, the most widely adopted modification strategies for enhancing the catalytic performance of MnO_x_‐based cathodes include morphological engineering to construct hierarchical porous architectures, defect engineering to boost catalytic activity, and composite strategies that integrate conductive scaffolds to improve electron transport [176, 177].

### 5.1 Construction of Porous Architectures

During discharge in LOBs, Li^+^, O_2_, and the electrolyte migrate to the O_2_ cathode, where they participate in the ORR at the solid‐liquid‐gas three‐phase interface, generating solid Li_2_O_2_. Once the pores of the O_2_ electrode become completely clogged by the discharge product, the reaction immediately terminates [178]. Thus, the designed cathode catalyst must not only provide continuous pathways and abundant active sites for Li^+^/O_2_/electrolyte transport and interfacial reactions, but also offer sufficient space to accommodate the insulating Li_2_O_2_.

Moreover, constructing porous architectures is also essential for maintaining cycling stability. Over short‐term cycling, large surface areas and hierarchical porosity help mitigate catalyst deactivation caused by the accumulation of side products, prevent pore blockage, and ensure that active sites remain accessible, thereby significantly improving reversibility.

**FIGURE 5:**
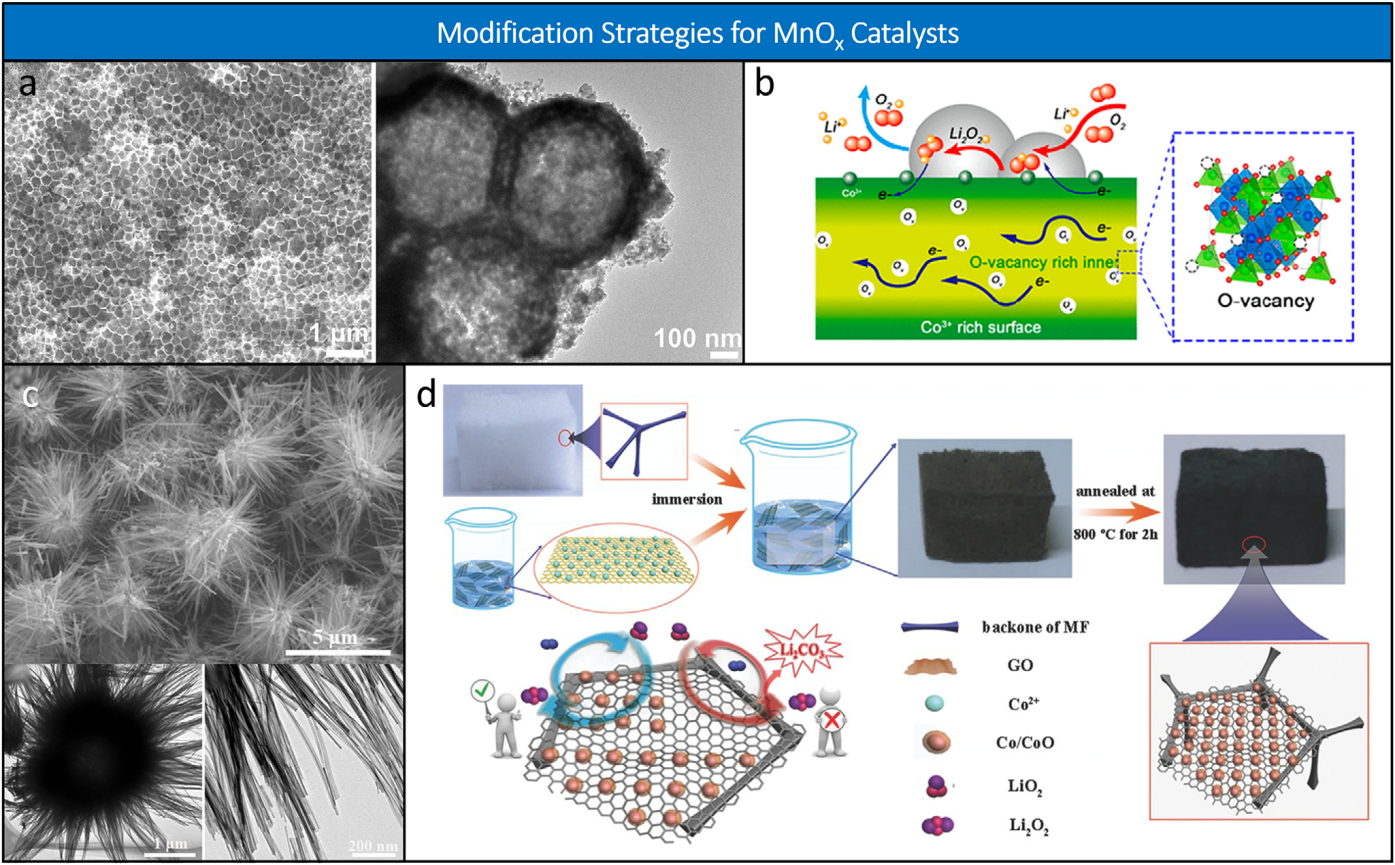
Representative modification strategies for improving MnO_x_‐based cathode catalysts in LOBs. (a) Porous CoMn_2_O_4_ spinel architectures prepared via hard‐template synthesis. Reproduced with permission [179]. Copyright 2024, Elsevier. (b) Schematic illustration showing the synergistic role of oxygen vacancies in regulating catalytic activity. Reproduced with permission [180]. Copyright 2017, American Chemical Society. (c) Morphologies of Ag‐doped α‐MnO_2_. Reproduced with permission [181]. Copyright 2020, Springer Nature. (d) Schematic diagram for the fabrication process of CMF‐G‐Co/CoO cathodes. Reproduced with permission [182]. Copyright 2016, Wiley.

Porous micro‐/nanotubes, nanoflowers, and various 3D architectures can be constructed through chemical synthesis routes [183, 184], while hard‐template removal strategies have been extensively employed for the fabrication of porous TM oxides and porous carbon materials (Figure 5a) [179, 185, 186]. The introduction of hierarchical porous structures is particularly beneficial for LOBs, as it can increase the accessibility of catalytic sites and provide sufficient space for the accommodation of discharge products. A sisal‐like Co_9_S_8_ catalyst developed for LOB applications exhibited an open fibrous architecture capable of promoting O_2_ diffusion and electrolyte infiltration while improving charge‐transfer kinetics [187]. In addition, the enlarged internal space effectively accommodated solid discharge products, alleviating pore blockage during cycling. Consequently, the assembled battery exhibited a low overpotential of only 0.57 V under a cutoff capacity of 1000 mAh g^−1^ and maintained stable cycling for approximately 100 cycles at 100 mA g^−1^.

Hard‐template engineering has also been applied to regulate pore structures with greater precision. Ordered mesoporous RuO_2_ with tunable pore sizes was synthesized using SiO_2_ spheres as a hard template and employed as a carbon‐free cathode for LOBs [188]. Systematic investigation revealed that both specific surface area and pore size strongly influence discharge capacity and round‐trip efficiency. Excessively small pores tend to become blocked rapidly by discharge products and restrict Li^+^/O_2_ transport, leading to reduced capacity and increased overpotential. In contrast, excessively large pores can weaken the structural integrity of mesoporous RuO_2_, resulting in accelerated capacity degradation during repeated cycling.

### 5.2 Defect‐Engineering Strategies

Studies have shown that introducing defect‐engineering strategies to modify the surfaces of metal‐based catalysts can substantially reduce reaction overpotentials and enhance cycling stability in LOBs [189, 190, 191, 192]. Crystal defects in solid materials are generally categorized as point defects (0D), line defects (1D), planar defects (2D), and bulk defects (3D) [193, 194, 195, 196, 197]. Among these, point‐defect engineering is the most widely adopted approach in electrode design [198]. By introducing vacancies, impurity atoms, or interstitial atoms into the crystal lattice, one can disrupt the periodic atomic arrangement, alter the local coordination environment and charge density, and ultimately regulate the surface physicochemical properties and electrical conductivity of the catalyst [199].

Common approaches for generating point defects include plasma etching, chemical reduction, thermal treatment, and heteroatom doping [200, 201]. Defect engineering has attracted considerable attention because the introduction of vacancies can effectively modify the local electronic environment and alter adsorption behavior during electrochemical reactions. Cation vacancies were introduced into cobalt oxides through regulation of the thermal‐treatment temperature of cobalt glycerolate precursors [202]. The generated Co vacancies induced delocalized charge redistribution on the oxide surface, which reduced the adsorption‐energy differences among reaction intermediates and facilitated more balanced catalytic pathways, thereby improving bifunctional catalytic activity.

In addition to cation vacancies, O‐vacancy engineering has also demonstrated a strong influence on catalytic performance. Nanosheets enriched with O vacancies were synthesized through thermal treatment under an O_2_ atmosphere (Figure 5b) [180]. The coexistence of O vacancies and metal ions with different valence states generated synergistic effects that promoted both the formation and decomposition of Li_2_O_2_. Such interactions facilitated a solution‐mediated growth mechanism and contributed to enhanced discharge capacity. Furthermore, introducing dopants into TM oxides, such as incorporating Ag and Ce into α‐MnO_2_, can simultaneously generate abundant vacancies and modify the electronic distribution of the host material (Figure 5c) [181, 203]. These structural and electronic changes can affect charge‐transfer behavior and active‐site characteristics, leading to improved intrinsic catalytic activity and electrical conductivity.

### 5.3 Composite Conductive Scaffolds

Integrating catalytic materials with conductive scaffolds is an effective strategy to enhance the electronic conductivity of metal oxides during charge/discharge processes and to expose a greater number of active catalytic sites. Among these scaffolds, carbon‐based materials have received particular attention owing to their low cost, excellent electrical conductivity, and structural versatility, ranging from graphene and CNTs to various porous carbons [204, 205, 206].

The construction of integrated electrode architectures has been widely explored to simultaneously enhance conductivity, structural stability, and catalytic activity in LOBs. A 3D hierarchical porous carbonized‐melamine‐foam/graphene/Co‐CoO composite cathode (CMF‐G‐Co/CoO, Figure 5d) was developed using high‐temperature carbonized melamine as the structural backbone [182]. The interconnected conductive framework and intrinsic mechanical elasticity of the material effectively improved electrode stability during repeated cycling. Electrochemical and structural characterizations further indicated that synergistic interactions between Co/CoO and graphene regulated the initial nucleation behavior of discharge products, promoting preferential Li_2_O_2_ growth on Co/CoO sites during the early discharge stage. Such selective deposition behavior effectively suppressed parasitic reactions occurring at the carbon/LiO_2_ interface.

The direct growth of active materials on conductive substrates provides another approach for constructing self‐supporting electrodes with enhanced transport properties. Mesoporous CoNi_2_S_4_ nanorod arrays grown directly on flexible carbon textiles through a two‐step hydrothermal process generated a self‐supporting cathode structure (CNS‐RAs/CT) [207]. The highly open architecture and large specific surface area increased the exposure of catalytic sites while supplying sufficient internal space for Li_2_O_2_ accommodation. These structural characteristics enabled the fabrication of a large‐area flexible pouch‐type LOB with an energy density of 484.29 Wh kg^−1^.

Metallic scaffolds, including stainless‐steel meshes and nickel foams, have also attracted increasing attention as conductive supports because of their high electrical conductivity and mechanical robustness. Multilayer N‐doped CNTs directly grown on stainless‐steel mesh produced a self‐supporting and flexible electrode (N‐CNTs@SS) with favorable physicochemical characteristics [208]. The intimate integration between the conductive scaffold and active components reduced interfacial resistance and facilitated charge transport, allowing the corresponding LOB to maintain stable cycling over 232 cycles.

### 5.4 Practical Relevance and System‐Level Considerations

Although substantial efforts have focused on improving the intrinsic catalytic activity of MnO_x_‐based cathodes, the practical performance of LOBs is strongly influenced by system‐level interactions involving electrolytes, redox mediators, carbon supports, and Li metal anodes [209]. Therefore, the design of MnO_x_ catalysts should not be considered independently but rather within an integrated battery environment.

The interactions between MnO_x_ catalysts and electrolyte systems are particularly important because reactive oxygen species and intermediate products generated during ORR/OER processes can initiate electrolyte decomposition [210]. The catalytic properties of MnO_x_ may influence adsorption energies and reaction pathways of oxygen intermediates, thereby affecting the formation of parasitic products such as Li_2_CO_3_ and other irreversible species. Rational regulation of surface electronic structures and defect sites may therefore help suppress undesirable side reactions and improve reaction selectivity.

In systems employing RMs, MnO_x_ catalysts may also influence mediator activity through interfacial electronic interactions [211]. Proper matching between MnO_x_ surface properties and mediator redox potentials could facilitate Li_2_O_2_ formation and decomposition pathways while reducing charge overpotentials. However, undesirable interactions may also lead to mediator degradation or additional parasitic reactions, indicating that catalyst–mediator compatibility requires further investigation. Furthermore, although MnO_x_ catalysts cannot directly resolve Li metal degradation issues, they may indirectly improve system stability by lowering charging overpotentials and reducing the generation of highly reactive intermediates [212]. Such effects can mitigate electrolyte decomposition and alleviate detrimental reactions occurring at the Li metal anode.

## 6 Summary and Perspective

Non‐aqueous LOBs possess an ultrahigh theoretical energy density, approaching that of gasoline (∼1700 Wh kg^−1^), and are therefore regarded as one of the most promising next‐generation energy‐storage technologies. However, their practical implementation remains severely constrained. The dominant discharge product, solid and electrically insulating Li_2_O_2_, is insoluble in typical organic electrolytes and exhibits strong oxidizing characteristics. These intrinsic properties result in limited discharge capacities, large overpotentials, and poor cycling stability.

**FIGURE 6:**
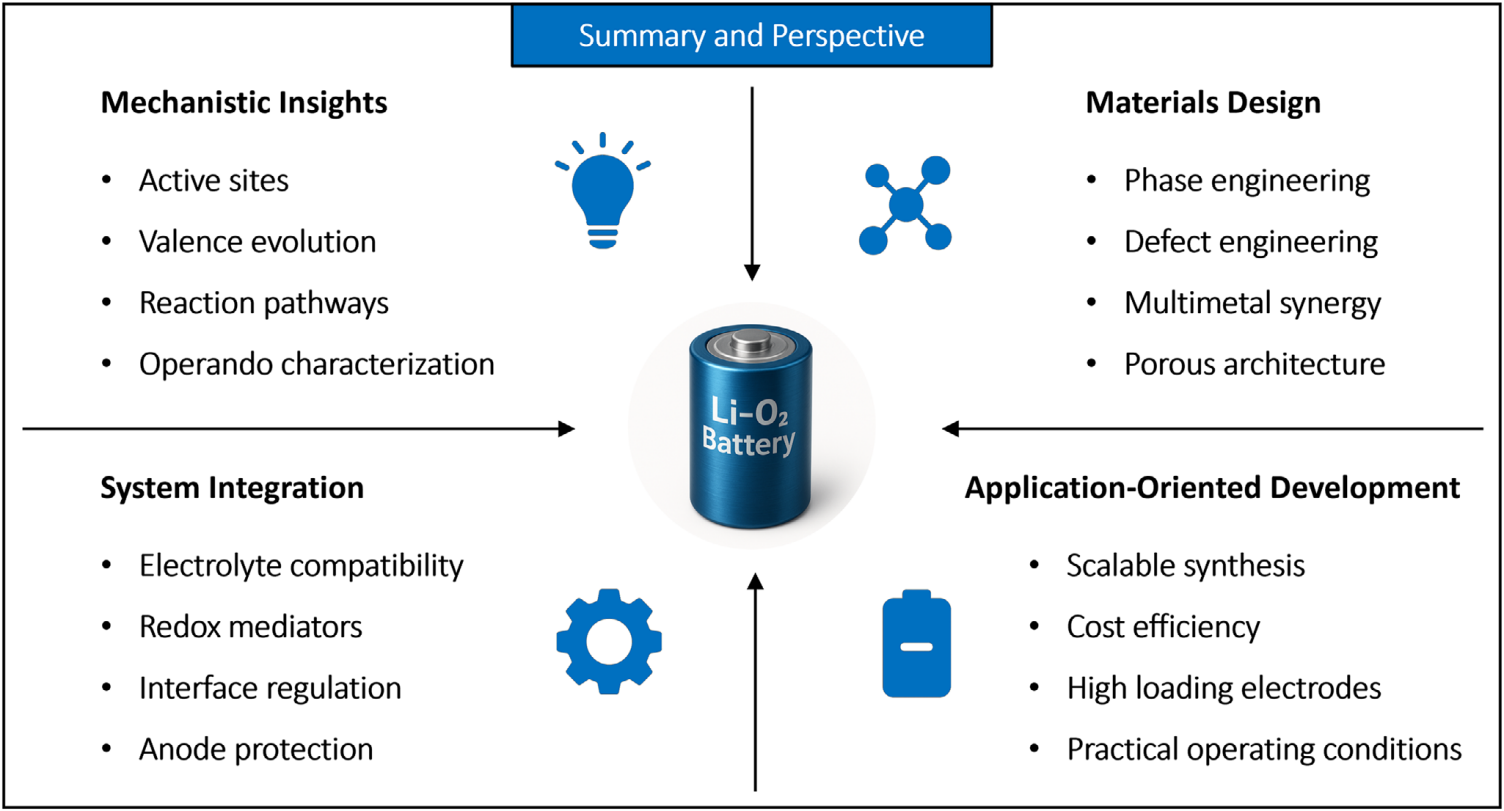
Summary and perspective of MnO_x_ materials for LOBs.

During discharge, continuous accumulation of Li_2_O_2_ progressively passivates catalytic sites and blocks ion and mass transport pathways. Meanwhile, its high oxidizing nature triggers persistent parasitic reactions with the electrolyte. During charging, the insulating nature of Li_2_O_2_ drastically hampers charge transfer, leading to severe polarization and large OER overpotentials. When the charging voltage exceeds ∼4.35 V, undesirable decomposition of both the electrolyte and carbon materials is accelerated, causing irreversible damage to the electrode architecture. As the locus of both the ORR/OER, the cathode catalyst plays a vital role in lowering reaction barriers and determining overall electrochemical performance (Figure 6).

An ideal cathode catalyst for high‐performance LOBs must simultaneously satisfy several criteria: i) High ORR and OER catalytic activity, enabling reduced overpotentials, complete decomposition of Li_2_O_2_, enhanced reaction reversibility, and improved energy efficiency; ii) Optimized micro‐/nanoporous architectures, capable of accommodating discharge products while maintaining channels for O_2_ diffusion and electrolyte infiltration, thereby enhancing capacity; iii) High electronic conductivity, which promotes rapid charge transport, suppresses polarization, and improves rate capability; iv) Abundant raw materials and low fabrication cost, which are essential for scaling toward practical applications.

MnO_x_, as a representative family of TM‐based catalysts, offer several advantages: abundant natural reserves, low cost, and intrinsic catalytic activity. Although their relatively low electronic conductivity limits reaction kinetics and leaves significant room for improvement, the rich Mn valence states and diverse crystalline phases endow them with exceptional tunability. Therefore, rational structural and compositional engineering of MnO_x_, guided by established catalyst‐design strategies, offers a promising pathway for improving LOB performance and advancing practical applications. Nevertheless, the practical deployment of MnO_x_‐based catalysts requires consideration beyond intrinsic catalytic activity. Future development should emphasize system‐level optimization involving catalyst–electrolyte compatibility, interactions with redox mediators, suppression of parasitic chemistry, and stabilization of Li metal anodes. Rather than acting as standalone solutions, MnO_x_‐based catalysts are expected to function as key components within integrated LOB systems employing stable electrolytes, efficient redox mediators, and protected Li metal architectures.

Moreover, future studies combining catalyst design with advanced operando characterization and theoretical calculations are expected to provide deeper insights into reaction pathways and dynamic catalyst evolution in MnO_x_‐based LOB systems. Such integrated approaches may facilitate the establishment of more reliable structure–activity relationships and accelerate the rational development of high‐performance MnO_x_ cathode catalysts for practical LOB applications.

## Conflicts of Interest

The authors declare no conflicts of interest.

## Data Availability

Data sharing not applicable to this article as no datasets were generated or analysed during the current study.

## References

[1] E. J. Askins , M. R. Zoric , M. Li , et al., “Triarylmethyl Cation Redox Mediators Enhance Li–O_2_ Battery Discharge Capacities,” Nature Chemistry 15, no. 9 (2023): 1247–1254. 10.1038/s41557-023-01268-0 37414882

[2] G. Zeng , Q. Sun , S. Horta , et al., “A Layered Bi_2_Te_3_ @PPy Cathode for Aqueous Zinc‐Ion Batteries: Mechanism and Application in Printed Flexible Batteries,” Advanced Materials 36, no. 1 (2024): 2305128. 10.1002/adma.202305128 37555532

[3] S. Ahn , C. Zor , S. Yang , et al., “Why Charging Li–Air Batteries With Current Low‐Voltage Mediators is Slow and Singlet Oxygen Does Not Explain Degradation,” Nature Chemistry 15, no. 7 (2023): 1022–1029. 10.1038/s41557-023-01203-3 37264102

[4] S. Wang , G. Zeng , Q. Sun , et al., “Flexible Electronic Systems via Electrohydrodynamic Jet Printing: A MnSe@rGo Cathode for Aqueous Zinc‐Ion Batteries,” ACS Nano 17, no. 14 (2023): 13256–13268. 37411016 10.1021/acsnano.3c00672

[5] H. Zhang , M. Sun , F. Sun , et al., “High‐Efficiency and High‐Capacity Aqueous Electrochromic Energy Storage Devices Enabled by Decoupled Titanium Oxide/Viologen Derivative Hybrid Materials,” Research 8 (2025): 0909. 41049609 10.34133/research.0909 PMC12491783

[6] R. C. McNulty , K. D. Jones , C. Holc , et al., “Hydroperoxide‐Mediated Degradation of Acetonitrile in the Lithium–Air Battery,” Advanced Energy Materials 13, no. 23 (2023): 2300579.

[7] Z. Xu , X. Guo , J. Wang , et al., “Restraining the Octahedron Collapse in Lithium and Manganese Rich NCM Cathode toward Suppressing Structure Transformation,” Advanced Energy Materials 12 (2022): 2201323.

[8] Q. Sun , J. Li , M. Yang , et al., “Carbon Microstructure Dependent Li‐Ion Storage Behaviors in SiO_x_/C Anodes,” Small 19, no. 25 (2023): 2300759. 10.1002/smll.202300759 36919820

[9] J. Li , K. V. Mejía‐Centeno , M. D. Khan , et al., “Beyond Imperfect Match: Silicon/Graphite Hybrid Anodes for High‐Energy–Density Lithium‐Ion Batteries,” Advanced Energy Materials 16, no. 10 (2026): 05674.

[10] C. Allard , “Li–Air Batteries Hitting the Road,” Nature Reviews Materials 8, no. 3 (2023): 145–145.

[11] M. Yu , C. Cao , Z. Sa , et al., “Liquid Metal Alchemy: Unlocking Self‐Healing Gallium‐Based Materials for Next‐Generation Electronics,” Materials Science and Engineering: R: Reports 166 (2025): 101073.

[12] G. Zeng , Y. Tian , M. D. Khan , et al., “Non‐Flammable Electrolytes for Safe Lithium‐, Sodium‐, and Potassium‐Ion Batteries,” Electron 3, no. 4 (2025): 70021.

[13] X. Zheng , K. V. Mejia‐Centeno , M. D. Khan , A. Cabot , and Q. Sun , “From Printed Architectures to Functional Batteries: Printing Technologies, Post‐Processing, and Sintering,” Advanced Materials Joining 1, no. 1 (2026): 2.

[14] G. Zeng , S. Horta , Q. Sun , et al., “Crystal Growth Engineering for Dendrite‐Free Zinc Metal Plating,” Advanced Materials (2025): 10906. 10.1002/adma.202510906 PMC13568996 41025826

[15] J. Li , G. Zeng , S. Horta , et al., “Crystallographic Engineering in Micron‐Sized SiO_x_ Anode Material toward Stable High‐Energy‐Density Lithium‐Ion Batteries,” ACS Nano 19, no. 16 (2025): 16096–16109. 40237414 10.1021/acsnano.5c03074

[16] S. Zhang , Q. Sun , P. R. Martínez‐Alanis , et al., “Towards Flame Retardant High‐Performance Solid‐State Lithium Metal Batteries: Poly(Ionic Liquid)‐Based Lithiophilic Ion‐Conductive Interfaces and Humidity Tolerant Binders,” Nano Energy 133 (2025): 110424.

[17] S. Wang , Y. Han , X. Ma , et al., “Na^+^‐Preintercalated Α‐MnO_2_ for Printed Electrodes of Aqueous Hybrid Supercapacitors,” Chemical Engineering Journal 531 (2026): 173921.

[18] Y. Yao , S. Wang , X. Ma , et al., “Iridium Cluster Decoration on Amorphous Cobalt Oxide‐Coated Carbon Nanotubes for High‐Performance Lithium‐Oxygen Battery Cathodes,” Small 21, no. 33 (2025): 2503521. 10.1002/smll.202503521 40545914

[19] S. Nam , M. Mahato , K. Matthews , et al., “Bimetal Organic Framework–Ti_3_C_2_T_x_ MXene with Metalloporphyrin Electrocatalyst for Lithium–Oxygen Batteries,” Advanced Functional Materials 33, no. 1 (2023): 2210702.

[20] S. Matsuda , M. Ono , H. Asahina , et al., “Chemical Crossover Accelerates Degradation of Lithium Electrode in High Energy Density Rechargeable Lithium–Oxygen Batteries,” Advanced Energy Materials 13, no. 11 (2023): 2203062.

[21] M. Longo , M. Gandolfo , C. Francia , S. Bodoardo , M. Sangermano , and J. Amici , “Gelatine Based Gel Polymer Electrolyte towards More Sustainable Lithium‐Oxygen Batteries,” Electrochimica Acta 466 (2023): 143026.

[22] E. L. Littauer and K. C. Tsai , “Anodic Behavior of Lithium in Aqueous Electrolytes: I . Transient Passivation,” Journal of the Electrochemical Society 123, no. 6 (1976): 771–776.

[23] K. M. Abraham and Z. Jiang , “A Polymer Electrolyte‐Based Rechargeable Lithium/Oxygen Battery,” Journal of the Electrochemical Society 143, no. 1 (1996): 1–5.

[24] T. Ogasawara , A. Débart , M. Holzapfel , P. Novák , and P. G. Bruce , “Rechargeable Li_2_O_2_ Electrode for Lithium Batteries,” Journal of the American Chemical Society 128, no. 4 (2006): 1390–1393. 16433559 10.1021/ja056811q

[25] P. Kichambare , S. Rodrigues , D. Firsich , et al., “Phthalocyanine‐Based Bifunctional Soluble Hybrid Catalyst for Rechargeable Lithium‐Oxygen Batteries,” Chemical Physics Letters 841 (2024): 141179.

[26] M. Jenkins , D. Dewar , M. Lagnoni , et al., “A High Capacity Gas Diffusion Electrode for Li–O_2_ Batteries,” Advanced Materials 36, no. 41 (2024): 2405715. 10.1002/adma.202405715 39101286

[27] S. Maiti , M. T. Curnan , S. Subhalaxmi , et al., “Adapting Single‐Atom Catalysts to Li–O_2_ Batteries: Enhancing Energy Storage,” Small 21, no. 35 (2025): 2505334. 10.1002/smll.202505334 40643008

[28] A. Narimani‐Qurtlar , A. Sayyah , S. Pakseresht , et al., “Investigating the Environmental Impacts of Lithium‐Oxygen Battery Cathode Production: A Comprehensive Assessment of the Effects Associated with Oxygen Cathode Manufacturing,” Journal of Cleaner Production 482 (2024): 144199.

[29] S. Barua , M. A. Rahman , K. C. Bhowmik , M. M. Billah , and H. M. A. R. Maruf , “Transition Metal Oxides as Promising Air‐Cathode Catalysts of Li‐Air Batteries: A State‐of‐the‐Art Review,” ChemistrySelect 10, no. 37 (2025): 04744.

[30] R. P. Rao , B. Ramasubramanian , R. Saritha , and S. Ramakrishna , “Microwave Assisted Synthesis for ϵ‐MnO_2_ Nanostructures on Ni foam as for Rechargeable Li–O_2_ Battery Applications,” Nano Express 4, no. 4 (2023): 045004.

[31] D. Choudhary , R. Bala , S. Yadav , and R. Dhiman , “MnO_2_ Nanowires as an Electrode Material for Gel Polymer Electrolyte‐Based Zn‐Air Battery,” Macromolecular Symposia 413, no. 1 (2024): 2300060.

[32] D. Cao , S. Zhang , F. Yu , Y. Wu , and Y. Chen , “Carbon‐Free Cathode Materials for Li−O_2_ Batteries,” Batteries & Supercaps 2, no. 5 (2019): 428–439.

[33] J.‐W. Jung , S.‐H. Cho , J. S. Nam , and I.‐D. Kim , “Current and Future Cathode Materials for Non‐Aqueous Li‐Air (O_2_) Battery Technology – A Focused Review,” Energy Storage Materials 24 (2020): 512–528.

[34] Z. Ma , X. Yuan , L. Li , et al., “A Review of Cathode Materials and Structures for Rechargeable Lithium–Air Batteries,” Energy & Environmental Science 8 (2015): 2144–2198.

[35] Z. Peng , S. A. Freunberger , Y. Chen , and P. G. Bruce , “A Reversible and Higher‐Rate Li‐O_2_ Battery,” Science 337, no. 6094 (2012): 563–566. 22821984 10.1126/science.1223985

[36] B. Horstmann , T. Danner , and W. G. Bessler , “Precipitation in Aqueous Lithium–Oxygen Batteries: A Model‐Based Analysis,” Energy & Environmental Science 6, no. 4 (2013): 1299.

[37] H. Kitaura and H. Zhou , “Electrochemical Performance of Solid‐State Lithium–Air Batteries Using Carbon Nanotube Catalyst in the Air Electrode,” Advanced Energy Materials 2, no. 7 (2012): 889–894.

[38] R. Black , B. Adams , and L. F. Nazar , “Non‐Aqueous and Hybrid Li‐O_2_ Batteries,” Advanced Energy Materials 2, no. 7 (2012): 801–815.

[39] M. Asmare Alemu , A. Ketema Worku , and M. Zegeye Getie , “Recent Advancement of Electrically Rechargeable Alkaline Metal‐Air Batteries for Future Mobility,” Results in Chemistry 6 (2023): 101048.

[40] Y. Hatakeyama , R. Oda , S. Yamamoto , and S. Shiraishi , “Exploring the Frontiers of Li–Air Battery Research: A Review of *In Situ*/*Operando* Measurement Techniques,” Carbon Reports 4, no. 2 (2025): 123–143.

[41] T. Zhang , N. Imanishi , Y. Takeda , and O. Yamamoto , “Aqueous Lithium/Air Rechargeable Batteries,” Chemistry Letters 40, no. 7 (2011): 668–673.

[42] Y. Liu , P. He , and H. Zhou , “Rechargeable Solid‐State Li–Air and Li–S Batteries: Materials, Construction, and Challenges,” Advanced Energy Materials 8, no. 4 (2018): 1701602.

[43] B. He , J. Wang , Y. Fan , et al., “Mesoporous CoO/Co–N–C Nanofibers as Efficient Cathode Catalysts for Li–O_2_ Batteries,” Journal of Materials Chemistry A 6, no. 39 (2018): 19075–19084.

[44] B. Ge , J. Wang , Y. Sun , J. Guo , C. Fernandez , and Q. Peng , “Heterojunction‐Composited Architecture for Li–O_2_ Batteries With Low Overpotential and Long‐Term Cyclability,” ACS Applied Energy Materials 3, no. 4 (2020): 3789–3797.

[45] T. Yang , Y. Xia , T. Mao , et al., “Phosphorus Vacancies and Heterojunction Interface as Effective Lithium‐Peroxide Promoter for Long‐Cycle Life Lithium–Oxygen Batteries,” Advanced Functional Materials 32, no. 49 (2022): 2209876.

[46] S. Zhang , Q. Sun , G. Hou , et al., “Boosting Fast Interfacial Li^+^ Transport in Solid‐State Li Metal Batteries via Ultrathin Al Buffer Layer,” Nano Research 16, no. 5 (2023): 6825–6832.

[47] Q. Sun , J. Li , C. Hao , and L. Ci , “Focusing on the Subsequent Coulombic Efficiencies of SiO_x_: Initial High‐Temperature Charge after over‐Capacity Prelithiation for High‐Efficiency SiO_x_‐Based Full‐Cell Battery,” ACS Applied Materials & Interfaces 14, no. 12 (2022): 14284–14292. 35298133 10.1021/acsami.2c01392

[48] S.‐Q. Li , J.‐N. Yang , K.‐X. Wang , and J.‐S. Chen , “Optimization Strategies for Cathode Materials in Lithium–Oxygen Batteries,” Accounts of Materials Research 5, no. 12 (2024): 1496–1506.

[49] A. Dutta , T. Kameda , J. Takada , Y. Nakajima , T. Morishita , and S. Matsuda , “Quantitative Porosity Engineering of Carbon Electrode in Lithium–Oxygen Batteries with Cell‐Level Gravimetric Energy Density over 1500 wh Kg^−1^ ,” Advanced Science 13, no. 1 (2026): 14406. 10.1002/advs.202514406 PMC12766985 41090490

[50] Q. Sun , D. Li , J. Cheng , et al., “Nitrogen‐Doped Carbon Derived from Pre‐Oxidized Pitch for Surface Dominated Potassium‐Ion Storage,” Carbon 155 (2019): 601–610.

[51] Q. Sun , G. Zeng , J. Li , et al., “Is Soft Carbon a More Suitable Match for SiO_x_ in Li‐Ion Battery Anodes?,” Small 19, no. 37 (2023): 2302644. 10.1002/smll.202302644 37144432

[52] S. Li , J. Xu , Z. Ma , et al., “NiMn_2_O_4_ as an Efficient Cathode Catalyst for Rechargeable Lithium–Air Batteries,” Chemical Communications 53, no. 58 (2017): 8164–8167. 28677707 10.1039/c7cc01995e

[53] L. Zou , J. Cheng , Y. Jiang , et al., “Spinel MnCo_2_O_4_ Nanospheres as an Effective Cathode Electrocatalyst for Rechargeable Lithium–Oxygen Batteries,” RSC Advances 6, no. 37 (2016): 31248–31255.

[54] Y. Zhu , S. Liu , C. Jin , S. Bie , R. Yang , and J. Wu , “MnO x Decorated CeO_2_ Nanorods as Cathode Catalyst for Rechargeable Lithium–Air Batteries,” Journal of Materials Chemistry A 3, no. 25 (2015): 13563–13567.

[55] S. Tong , M. Zheng , Y. Lu , et al., “Mesoporous NiO With a Single‐Crystalline Structure Utilized as a Noble Metal‐Free Catalyst for Non‐Aqueous Li–O_2_ Batteries,” Journal of Materials Chemistry A 3, no. 31 (2015): 16177–16182.

[56] R. Peng , Y. Li , T. Liu , et al., “Reduced Graphene Oxide/SnO_2_@Au Heterostructure for Enhanced Ammonia Gas Sensing,” Chemical Physics Letters 737 (2019): 136829.

[57] J. Li , Y. Li , J. Cheng , et al., “A Graphene Oxide Coated Sulfide‐Based Solid Electrolyte for Dendrite‐Free Lithium Metal Batteries,” Carbon 177 (2021): 52–59.

[58] Q. Sun , M. Yang , G. Zeng , et al., “Insights Into the Potassium Ion Storage Behavior and Phase Evolution of a Tailored Yolk–Shell SnSe@C Anode,” Small 18, no. 39 (2022): 2203459. 10.1002/smll.202203459 36026577

[59] Q. Sun , D. Li , L. Dai , Z. Liang , and L. Ci , “Structural Engineering of SnS_2_ Encapsulated in Carbon Nanoboxes for High‐Performance Sodium/Potassium‐Ion Batteries Anodes,” Small 16, no. 45 (2020): 2005023. 10.1002/smll.202005023 33079488

[60] J. Gao , X. Cai , J. Wang , M. Hou , L. Lai , and L. Zhang , “Recent Progress in Hierarchically Structured O_2_‐Cathodes for Li‐O_2_ Batteries,” Chemical Engineering Journal 352 (2018): 972–995.

[61] H.‐D. Lim , K.‐Y. Park , H. Song , et al., “Enhanced Power and Rechargeability of a Li−O_2_ Battery Based on a Hierarchical‐Fibril CNT Electrode,” Advanced Materials 25, no. 9 (2013): 1348–1352. 23255225 10.1002/adma.201204018

[62] M. Lee , Y. Yoo , J. H. Kwak , et al., “Effect of Surface Characteristics of Carbon Host on Electrochemical Performance of Nonaqueous Li–O_2_ Batteries,” Chemical Engineering Journal 412 (2021): 128549.

[63] M. He , P. Zhang , L. Liu , B. Liu , and S. Xu , “Hierarchical Porous Nitrogen Doped Three‐Dimensional Graphene as a Free‐Standing Cathode for Rechargeable Lithium‐Oxygen Batteries,” Electrochimica Acta 191 (2016): 90–97.

[64] D. Y. Kim , M. Kim , D. W. Kim , et al., “Graphene Paper With Controlled Pore Structure for High‐Performance Cathodes in Li–O_2_ Batteries,” Carbon 100 (2016): 265–272.

[65] J. Kang , D. Y. Kim , J. Suk , et al., “Enhanced Energy and O_2_ Evolution Efficiency Using an In Situ Electrochemically N‐Doped Carbon Electrode in Non‐Aqueous Li–O_2_ Batteries,” Journal of Materials Chemistry A 3, no. 37 (2015): 18843–18846.

[66] M. M. Ottakam Thotiyl , S. A. Freunberger , Z. Peng , and P. G. Bruce , “The Carbon Electrode in Nonaqueous Li–O_2_ Cells,” Journal of the American Chemical Society 135, no. 1 (2013): 494–500. 23190204 10.1021/ja310258x

[67] S. Liu , Z. Wang , C. Yu , et al., “Free‐Standing, Hierarchically Porous Carbon Nanotube Film as a Binder‐Free Electrode for High‐Energy Li–O_2_ Batteries,” Journal of Materials Chemistry A 1, no. 39 (2013): 12033.

[68] Y. Dou , X.‐G. Wang , D. Wang , et al., “Tuning the Structure and Morphology of Li_2_O_2_ by Controlling the Crystallinity of Catalysts for Li‐O_2_ Batteries,” Chemical Engineering Journal 409 (2021): 128145.

[69] B. J. Bergner , A. Schürmann , K. Peppler , A. Garsuch , and J. Janek , “Tempo: A Mobile Catalyst for Rechargeable Li‐O_2_ Batteries,” Journal of the American Chemical Society 136, no. 42 (2014): 15054–15064. 25255228 10.1021/ja508400m

[70] K. Liao , T. Zhang , Y. Wang , et al., “Nanoporous Ru as a Carbon‐ and Binder‐Free Cathode for Li–O_2_ Batteries,” Chemsuschem 8, no. 8 (2015): 1429–1434. 25809196 10.1002/cssc.201403371

[71] H. T. Bui , D. Y. Kim , D. W. Kim , J. Suk , and Y. Kang , “Carbon Nanofiber@Platinum by a Coaxial Electrospinning and Their Improved Electrochemical Performance as a Li−O_2_ Battery Cathode,” Carbon 130 (2018): 94–104.

[72] T. Zhang , B. Zou , X. Bi , et al., “Selective Growth of a Discontinuous Subnanometer Pd Film on Carbon Defects for Li–O_2_ Batteries,” ACS Energy Letters 4, no. 12 (2019): 2782–2786.

[73] K. Li , H. Dong , Y. Wang , Y. Yin , and S. Yang , “Preparation of Low‐Load Au‐Pd Alloy Decorated Carbon Fibers Binder‐Free Cathode for Li‐O_2_ Battery,” Journal of Colloid and Interface Science 579 (2020): 448–454. 32623116 10.1016/j.jcis.2020.06.084

[74] L. Wei , Y. Ma , Y. Gu , et al., “Ru‐Embedded Highly Porous Carbon Nanocubes Derived from Metal–Organic Frameworks for Catalyzing Reversible Li_2_O_2_ Formation,” Acs Applied Materials & Interfaces 13, no. 24 (2021): 28295–28303. 34102061 10.1021/acsami.1c06572

[75] S. Kumar , S. Chinnathambi , and N. Munichandraiah , “Ir Nanoparticles‐Anchored Reduced Graphene Oxide as a Catalyst for Oxygen Electrode in Li–O_2_ Cells,” New Journal of Chemistry 39, no. 9 (2015): 7066–7075. 10.1039/c4cp02858a 25242373

[76] Z. Q. Peng , S. A. Freunberger , Y. H. Chen , and P. G. Bruce , “A Reversible and Higher‐Rate Li‐O_2_ Battery,” Science 337, no. 6094 (2012): 563–566. 22821984 10.1126/science.1223985

[77] J.‐J. Xu , Z.‐L. Wang , D. Xu , L.‐L. Zhang , and X.‐B. J. N. C. Zhang , “Tailoring Deposition and Morphology of Discharge Products towards High‐Rate and Long‐Life Lithium‐Oxygen Batteries,” Nature Communications 4, no. 1 (2013): 1–10. 10.1038/ncomms3438 24052126

[78] J. Shen , H. Wu , W. Sun , et al., “In‐Situ Nitrogen‐Doped Hierarchical Porous Hollow Carbon Spheres Anchored with Iridium Nanoparticles as Efficient Cathode Catalysts for Reversible Lithium‐Oxygen Batteries,” Chemical Engineering Journal 358 (2019): 340–350.

[79] J. Li , S. Ding , S. Zhang , et al., “Catalytic Redox Mediators for Non‐Aqueous Li‐O_2_ Battery,” Energy Storage Materials 43 (2021): 97–119.

[80] H.‐S. Kim , B. Kim , H. Park , J. Kim , and W.‐H. Ryu , “Auto‐Oxygenated Porphyrin‐Derived Redox Mediators for High‐Performance Lithium Air‐Breathing Batteries,” Advanced Energy Materials 12, no. 7 (2022): 2103527.

[81] J. N. Lai , Y. Xing , N. Chen , L. Li , F. Wu , and R. J. Chen , “Electrolytes for Rechargeable Lithium–Air Batteries,” Angewandte Chemie International Edition 59, no. 8 (2020): 2974–2997. 31124264 10.1002/anie.201903459

[82] M. J. Lacey , J. T. Frith , and J. R. Owen , “A Redox Shuttle to Facilitate Oxygen Reduction in the Lithium Air Battery,” Electrochemistry Communications 26 (2013): 74–76.

[83] X. Gao , Y. Chen , L. Johnson , and P. G. Bruce , “Promoting Solution Phase Discharge in Li‐O_2_ Batteries Containing Weakly Solvating Electrolyte Solutions,” Nature Materials 15, no. 8 (2016): 882. 27443910 10.1038/nmat4691

[84] Z. Z. Shen , S. Y. Lang , C. Zhou , R. Wen , and L. J. Wan , “In Situ Realization of Water‐Mediated Interfacial Processes at Nanoscale in Aprotic Li‐O_2_ Batteries,” Advanced Energy Materials 10, no. 46 (2020): 2002339.

[85] Y. Dou , Z. Xie , Y. Wei , Z. Peng , and Z. Zhou , “Redox Mediators for High‐Performance Lithium–Oxygen Batteries,” National Science Review 9, no. 4 (2022): nwac040. 35548381 10.1093/nsr/nwac040 PMC9084180

[86] A. Y. Tesio , D. Blasi , M. Olivares‐Marin , I. Ratera , D. Tonti , and J. Veciana , “Organic Radicals for the Enhancement of Oxygen Reduction Reaction in Li–O_2_ Batteries,” Chemical Communications 51, no. 99 (2015): 17623–17626. 26488114 10.1039/c5cc07242e

[87] Y. Zhang , L. Wang , X. Zhang , L. Guo , Y. Wang , and Z. Peng , “High‐Capacity and High‐Rate Discharging of a Coenzyme Q 10 ‐Catalyzed Li–O_2_ Battery,” Advanced Materials 30, no. 5 (2018): 1705571. 10.1002/adma.201705571 29226435

[88] T. Homewood , J. T. Frith , J. P. Vivek , et al., “Using Polyoxometalates to Enhance the Capacity of Lithium–Oxygen Batteries,” Chemical Communications 54, no. 69 (2018): 9599–9602. 30094429 10.1039/c8cc03832e

[89] H.‐D. Lim , B. Lee , Y. Zheng , et al., “Rational Design of Redox Mediators for Advanced Li–O_2_ Batteries,” Nature Energy 1, no. 6 (2016): 16066.

[90] Y. Chen , S. A. Freunberger , Z. Peng , O. Fontaine , and P. G. Bruce , “Charging a Li–O_2_ Battery Using a Redox Mediator,” Nature Chemistry 5, no. 6 (2013): 489–494. 10.1038/nchem.1646 23695630

[91] Y. Yoon , S. Shin , and M. W. Shin , “Ammonium Ionic Liquid‐Functionalized Phenothiazine as a New Redox Mediator for High Chemical Stability on the Anode Surface in Lithium–Air Batteries,” ACS Applied Materials & Interfaces 14, no. 3 (2022): 4220–4229. 35005895 10.1021/acsami.1c22261

[92] W.‐J. Kwak , S.‐J. Park , H.‐G. Jung , and Y.‐K. Sun , “Optimized Concentration of Redox Mediator and Surface Protection of Li Metal for Maintenance of High Energy Efficiency in Li–O_2_ Batteries,” Advanced Energy Materials 8, no. 9 (2018): 1702258.

[93] L. Wang , J. Pan , Y. Zhang , X. Cheng , L. Liu , and H. Peng , “A Li–Air Battery with Ultralong Cycle Life in Ambient Air,” Advanced Materials 30, no. 3 (2018): 1704378. 10.1002/adma.201704378 29194803

[94] Y. Ko , K. Kim , J. Yoo , et al., “Redox Mediators for Oxygen Reduction Reactions in Lithium–Oxygen Batteries: Governing Kinetics and Its Implications,” Energy & Environmental Science 16, no. 11 (2023): 5525–5533.

[95] W. Li , Y. Ma , C. Sheng , et al., “Designing Multifunctional Catalysts for Lithium–Oxygen Batteries via an Amine–Epoxide Ring‐Opening Reaction,” Journal of the American Chemical Society 148, no. 10 (2026): 10880–10890. 41776997 10.1021/jacs.5c21190

[96] R. Bi , G. X. Liu , C. Zeng , X. P. Wang , L. Zhang , and S. Z. Qiao , “3D Hollow α‐MnO_2_ Framework as an Efficient Electrocatalyst for Lithium–Oxygen Batteries,” Small 15, no. 10 (2019): 7. 10.1002/smll.201804958 30714342

[97] K. R. Yoon , K. Shin , J. Park , et al., “Brush‐Like Cobalt Nitride Anchored Carbon Nanofiber Membrane: Current Collector‐Catalyst Integrated Cathode for Long Cycle Li–O_2_ Batteries,” ACS Nano 12, no. 1 (2018): 128–139. 29178775 10.1021/acsnano.7b03794

[98] X. X. Wang , J. H. Tian , X. Cheng , R. Na , D. D. Wang , and Z. Q. Shan , “Chitosan‐Induced Synthesis of Hierarchical Flower Ridge‐Like MoS_2_/N‐Doped Carbon Composites with Enhanced Lithium Storage,” ACS Applied Materials & Interfaces 10, no. 42 (2018): 35953–35962. 30264988 10.1021/acsami.8b11593

[99] J. Pan , X. L. Tian , S. Zaman , et al., “Recent Progress on Transition Metal Oxides as Bifunctional Catalysts for Lithium‐Air and Zinc‐Air Batteries,” Batteries & Supercaps 2, no. 4 (2019): 336–347.

[100] C. Sun , F. Xiao , D. Wu , et al., “Co Nanoparticles Embedded into Leaf‐Like Porous Carbon as a Promising Cathode Catalyst for Li‐O_2_ Batteries,” Journal of Electroanalytical Chemistry 944 (2023): 117663.

[101] R. Bi , G. Liu , C. Zeng , X. Wang , L. Zhang , and S. Z. Qiao , “3D Hollow α‐MnO_2_ Framework as an Efficient Electrocatalyst for Lithium–Oxygen Batteries,” Small 15, no. 10 (2019): 1804958. 10.1002/smll.201804958 30714342

[102] W. Zhao , X. Li , R. Yin , et al., “Urchin‐Like NiO–NiCo_2_O_4_ Heterostructure Microsphere Catalysts for Enhanced Rechargeable Non‐Aqueous Li–O_2_ Batteries,” Nanoscale 11 (2019): 50–59. 10.1039/c8nr08457b 30534796

[103] S. Jayabal , J. Wu , J. Y. Chen , D. S. Geng , and X. B. Meng , “Metallic 1T‐MoS_2_ Nanosheets and Their Composite Materials: Preparation, Properties and Emerging Applications,” Materials Today Energy 10 (2018): 264–279.

[104] L. Feng , Y. Li , L. Sun , H. Mi , X. Ren , and P. Zhang , “Heterostructured CoO‐Co_3_O_4_ Nanoparticles Anchored on Nitrogen‐Doped Hollow Carbon Spheres as Cathode Catalysts for Li–O_2_ Batteries,” Nanoscale 11, no. 31 (2019): 14769–14776. 31348479 10.1039/c9nr03521d

[105] L. Li , M. Hua , J. Li , et al., “Tuning Dual Catalytic Active Sites of Pt Single Atoms Paired with High‐Entropy Alloy Nanoparticles for Advanced Li‐O_2_ Batteries,” ACS Nano 19, no. 4 (2025): 4391–4402. 39824779 10.1021/acsnano.4c12499

[106] Y. Zhou , G. Hong , and W. Zhang , “Nanoengineering of Cathode Catalysts for Li–O_2_ Batteries,” ACS Nano 18, no. 26 (2024): 16489–16504. 38899523 10.1021/acsnano.4c04420

[107] S. Cho , L. Lyu , and Y.‐M. Kang , “Molecularly Programmed MOF Electrodes Enable Spatial Regulation of Triple‐Phase Boundaries in Li–O_2_ Batteries,” Nano Letters 26, no. 20 (2026): 6619–6626. 42119132 10.1021/acs.nanolett.6c00945

[108] C. Dang , Q. Mu , X. Xie , et al., “Recent Progress in Cathode Catalyst for Nonaqueous Lithium Oxygen Batteries: A Review,” Advanced Composites and Hybrid Materials 5, no. 2 (2022): 606–626.

[109] Z. Xu , Y. Yuan , Q. Tang , et al., “Facile Construction of a Multilayered Interface for a Durable Lithium‐Rich Cathode,” Carbon Energy 5, no. 9 (2023): 332.

[110] Z. Xu , L. Ci , Y. Yuan , et al., “Potassium Prussian Blue‐Coated Li‐Rich Cathode with Enhanced Lithium Ion Storage Property,” Nano Energy 75 (2020): 104942.

[111] Y. Feng , Y. Yao , S. Wang , et al., “Role of Hetero‐Doped Reduced Graphene Oxide in Suppressing Elemental Dissolution in Manganese Selenide Cathode for Aqueous Zinc‐Ion Batteries,” Chemsuschem 18, no. 8 (2025): 202402101. 10.1002/cssc.202402101 39624953

[112] L. Chen , G. Zeng , Q. Sun , et al., “K‐Ion Preintercalated MnO_2_ Nanorods as a High‐Rate Cathode Material for Aqueous Zinc‐Ion Batteries,” Ceramics International 50, no. 23 (2024): 52103–52109.

[113] G. Wang , X. Hu , J. Wang , et al., “Toward Practical Photo‐Assisted Li‐O_2_ Batteries: A Four‐Electron Pathway Enabled by Ru‐Doped Β‐MnO_2_ ,” Advanced Materials 37, no. 34 (2025): 2507891. 10.1002/adma.202507891 40484901

[114] S. Wang , Q. Chen , T. Gao , and Y. Zhou , “Z‐Scheme in_2_S_3_/MnO_2_/Biocl Heterojunction Photo‐Enhanced High‐Performance Lithium‐Oxygen Batteries,” Journal of Materials Science & Technology 215 (2025): 1–14.

[115] Q. Kang , H. Sun , and T. Zhang , “Effect of Ab‐RuO_2_/MnO_2_ Bifunctional Electrocatalyst on the Performance of Li‐O_2_ Batteries,” ChemistrySelect 10, no. 26 (2025): 02240.

[116] S.‐Z. Zhao , L.‐N. Song , M.‐R. Xie , et al., “Asymmetric Catalytic Site Driving LiOH Chemistry for Li–O_2_ Batteries Based on Cationic Vacancy‐Derived Single‐Atom Spinel,” ACS Catalysis 14 (2024): 7332–7344.

[117] Y. Zhu , J. Gao , Z. Wang , et al., “O_4_ Electrodes for High‐Performance Lithium‐Oxygen Batteries,” ChemPhysMater 3, no. 1 (2024): 94–102.

[118] P. Kannan , S. Guddehalli Chandrappa , R. K. Gupta , and A. S. Prakash , “Enhanced Oxygen Redox Kinetics and Stability in Li−O_2_ Batteries Using Trimetallic MnCo_2‐x_ Cu_x_O_4_ Spinel Catalysts,” Batteries & Supercaps 8, no. 6 (2025): 202400615.

[119] S. Yin , D. Yan , Y. Yan , et al., “Engineering of MnTe/MnO Heterostructures with Interfacial Electric Field Modulation for Efficient and Durable Li–O_2_ Batteries,” Small 20, no. 50 (2024): 2406525. 10.1002/smll.202406525 39308256

[120] J. Chen , R. Li , B. Li , et al., “Engineering Dual‐Crystal Configurations in Perovskite Oxides Boosts Electrocatalysis of Lithium–Oxygen Batteries,” Journal of Colloid and Interface Science 657 (2024): 384–392. 38056043 10.1016/j.jcis.2023.11.179

[121] D. Du , H. He , R. Zheng , et al., “Single‐Atom Immobilization Boosting Oxygen Redox Kinetics of High‐Entropy Perovskite Oxide Toward High‐Performance Lithium‐Oxygen Batteries,” Advanced Energy Materials 14, no. 17 (2024): 2304238.

[122] Z. Liu , H. Xu , X. Wang , G. Tian , D. Du , and C. Shu , “Strain‐Rich High‐Entropy Perovskite Oxide of (La_0.8_Sr_0.2_)(Mn_0.2_Fe_0.2_Cr_0.2_Co_0.2_Ni_0.2_)O_3_ for Durable and Effective Catalysis of Oxygen Redox Reactions in Lithium‐Oxygen Battery,” Battery Energy 3, no. 2 (2024): 20230053.

[123] Y. Zhu , Z. Wang , J. Gao , et al., “2D Co‐Doped MnCr_2_O_4_ Nanosheets as Efficient Bifunctional Cathode Materials for Long‐Life Li–O_2_ Batteries,” Inorganic Chemistry Frontiers 10, no. 14 (2023): 4252–4265.

[124] H. Lv , C. Fan , X. Xu , C. Zhao , and J. Long , “Design of Chromium‐Doped Spinel Mn_3_O_4_ Modulated Electronic Structure as an Efficient Catalyst for Li‐O_2_ Batteries,” Journal of Alloys and Compounds 953 (2023): 170130.

[125] L. Zhao , J. Feng , A. Abbas , C. Wang , and H. Wang , “MOF‐Derived Mn_2_O_3_ Nanocage With Oxygen Vacancies as Efficient Cathode Catalysts for Li–O_2_ Batteries,” Small 19, no. 41 (2023): 2302953. 10.1002/smll.202302953 37300361

[126] R. Palani , Y.‐S. Wu , S.‐H. Wu , et al., “Fascinating Bifunctional Electrocatalytic Activity via a Mesoporous Structured FeMnO_3_@ZrO_2_ Matrix as an Efficient Cathode for Li–O_2_ Batteries,” ACS Applied Energy Materials 6, no. 9 (2023): 4734–4747.

[127] M. Li , E. Li , J. Yi , L. Xiao , and J. Liu , “A Grafted Hybrid Nanosheet Array Architecture Enables Efficient Bifunctional Oxygen Catalytic Electrodes for Rechargeable Lithium−Oxygen Batteries,” Advanced Sustainable Systems 8, no. 5 (2024): 2300510.

[128] R. Huang , J. Chen , X. Chen , et al., “Synergized Oxygen Vacancies With Mn_2_O_3_@CeO_2_ Heterojunction as High Current Density Catalysts for Li–O_2_ Batteries,” Chinese Journal of Structural Chemistry 42, no. 11 (2023): 100171.

[129] Z. Xie , Z. Lu , J. Wang , Y. Qu , and L. Duan , “The Surface Electronic Structure Reconstruction of Co_2.85_Mn_0.15_O_4_ with High Active Sites for High‐Efficient Lithium–Oxygen Batteries,” ACS Sustainable Chemistry & Engineering 11, no. 17 (2023): 6698–6709.

[130] B. Liu , X. Liu , C. Wei , et al., “Constructing Asymmetrical Dual Catalytic Sites in Manganese Oxides Enables Fast and Stable Lithium–Oxygen Catalysis,” Journal of Materials Chemistry A 11, no. 3 (2023): 1188–1198.

[131] B. Liu , Y. Sun , L. Liu , S. Xu , and X. Yan , “Advances in Manganese‐Based Oxides Cathodic Electrocatalysts for Li–Air Batteries,” Advanced Functional Materials 28, no. 15 (2018): 1704973.

[132] K. R. Yoon , G. Y. Lee , J. W. Jung , N. H. Kim , S. O. Kim , and I. D. Kim , “One‐Dimensional RuO_2_/Mn_2_O_3_ Hollow Architectures as Efficient Bifunctional Catalysts for Lithium–Oxygen Batteries,” Nano Letters 16, no. 3 (2016): 2076–2083. 26821307 10.1021/acs.nanolett.6b00185

[133] S.‐L. Chou , J.‐Z. Wang , S.‐Y. Chew , H.‐K. Liu , and S.‐X. Dou , “Electrodeposition of MnO_2_ Nanowires on Carbon Nanotube Paper as Free‐Standing, Flexible Electrode for Supercapacitors,” Electrochemistry Communications 10, no. 11 (2008): 1724–1727.

[134] Y. Sun , H.‐W. Lee , Z. W. Seh , et al., “High‐Capacity Battery Cathode Prelithiation to Offset Initial Lithium Loss,” Nature Energy 1, no. 1 (2016): 15008.

[135] C. Ling and F. Mizuno , “Capture Lithium in αMnO_2_: Insights from First Principles,” Chemistry of Materials 24, no. 20 (2012): 3943–3951.

[136] Y. Meng , W. Song , H. Huang , Z. Ren , S. Y. Chen , and S. L. Suib , “Structure–Property Relationship of Bifunctional MnO_2_ Nanostructures: Highly Efficient, Ultra‐Stable Electrochemical Water Oxidation and Oxygen Reduction Reaction Catalysts Identified in Alkaline Media,” Journal of the American Chemical Society 136, no. 32 (2014): 11452–11464. 25058174 10.1021/ja505186m

[137] A. Debart , A. J. Paterson , J. Bao , and P. G. Bruce , “α‐MnO_2_ Nanowires: A Catalyst for the O_2_ Electrode in Rechargeable Lithium Batteries,” Angewandte Chemie International Edition 47, no. 24 (2008): 4521–4524. 18461594 10.1002/anie.200705648

[138] E. M. Benbow , S. P. Kelly , L. Zhao , J. W. Reutenauer , and S. L. Suib , “Oxygen Reduction Properties of Bifunctional Α‐Manganese Oxide Electrocatalysts in Aqueous and Organic Electrolytes,” The Journal of Physical Chemistry C 115, no. 44 (2011): 22009–22017.

[139] S. Chen , G. Liu , H. Yadegari , H. Wang , and S. Z. Qiao , “Three‐Dimensional MnO_2_ Ultrathin Nanosheet Aerogels for High‐Performance Li–O_2_ Batteries,” Journal of Materials Chemistry A 3, no. 6 (2015): 2559–2563.

[140] W.‐B. Luo , S.‐L. Chou , W. Jia‐Zhao , Y.‐C. Zhai , and H.‐K. Liu , “A Facile Approach to Synthesize Stable CNTs@MnO Electrocatalyst for High Energy Lithium Oxygen Batteries,” Scientific Reports 5, no. 1 (2015): 8012. 25634100 10.1038/srep08012 PMC4311244

[141] L. Han , S. Dong , and E. Wang , “Transition‐Metal (Co, Ni, and Fe)‐Based Electrocatalysts for the Water Oxidation Reaction,” Advanced Materials 28, no. 42 (2016): 9266–9291. 27569575 10.1002/adma.201602270

[142] K.‐N. Jung , J.‐I. Lee , S. Yoon , et al., “Manganese Oxide/Carbon Composite Nanofibers: Electrospinning Preparation and Application as a Bi‐Functional Cathode for Rechargeable Lithium–Oxygen Batteries,” Journal of Materials Chemistry 22, no. 41 (2012): 21845.

[143] S. Peng , L. Li , Y. Hu , et al., “Fabrication of Spinel One‐Dimensional Architectures by Single‐Spinneret Electrospinning for Energy Storage Applications,” ACS Nano 9, no. 2 (2015): 1945–1954. 25602640 10.1021/nn506851x

[144] G.‐H. Lee , S. Lee , J.‐C. Kim , D. W. Kim , Y. Kang , and D.‐W. Kim , “MnMoO_4_ Electrocatalysts for Superior Long‐Life and High‐Rate Lithium‐Oxygen Batteries,” Advanced Energy Materials 7, no. 6 (2017): 1601741.

[145] X. P. Han , Y. X. Hu , J. G. Yang , F. Y. Cheng , and J. Chen , “Porous Perovskite CaMnO_3_ as an Electrocatalyst for Rechargeable Li–O_2_ Batteries,” Chemical Communications 50, no. 12 (2014): 1497–1499. 24366540 10.1039/c3cc48207c

[146] D. Chen , C. Chen , Z. M. Baiyee , Z. Shao , and F. Ciucci , “Nonstoichiometric Oxides as Low‐Cost and Highly‐Efficient Oxygen Reduction/Evolution Catalysts for Low‐Temperature Electrochemical Devices,” Chemical Reviews 115, no. 18 (2015): 9869–9921. 26367275 10.1021/acs.chemrev.5b00073

[147] A. Débart , J. Bao , G. Armstrong , and P. G. Bruce , “An O_2_ Cathode for Rechargeable Lithium Batteries: The Effect of a Catalyst,” Journal of Power Sources 174, no. 2 (2007): 1177–1182.

[148] J. J. Xu , D. Xu , Z. L. Wang , H. G. Wang , L. L. Zhang , and X. B. Zhang , “Synthesis of Perovskite‐Based Porous La_0.75_Sr_0.25_MnO_3_ Nanotubes as a Highly Efficient Electrocatalyst for Rechargeable Lithium–Oxygen Batteries,” Angewandte Chemie International Edition 52, no. 14 (2013): 3887–3890. 23447093 10.1002/anie.201210057

[149] V. Celorrio , L. Calvillo , E. Dann , et al., “Oxygen reduction reaction at La_x_Ca_1−x_ MnO_3_ Nanostructures: Interplay Between A‐Site Segregation and B‐Site Valency,” Catalysis Science & Technology 6, no. 19 (2016): 7231–7238.

[150] D. Li , M. Görlin , R. Li , et al., “Improving Cycling Stability in Li‐O_2_ Batteries through in‐Situ SEI Formation Using 4‐Fluoro‐2‐Iodoaniline: Interface Morphology and Kinetic Insights via Operando Xrd and X‐Ray Ct,” Journal of Power Sources 676 (2026): 239845.

[151] H. Kim , H. Lee , W. Choi , et al., “Operando Observation of the De‐Evolution/Evolution Process of Hydrated LiOH in Moisture‐Assisted Li–O_2_ Batteries,” ACS Applied Materials & Interfaces 15, no. 24 (2023): 29120–29126. 37294066 10.1021/acsami.3c03661

[152] Y. Hatakeyama , H. Naito , S. Yamamoto , et al., “Fe‐Doped MnO_2_ Catalysts for Li–O_2_ Batteries: Mechanistic Insights into Durability Enhancement via Operando XAFS,” ACS Applied Energy Materials 9, no. 1 (2026): 251–260.

[153] P. Zhang , B. Han , X. Yang , et al., “Revealing the Intrinsic Atomic Structure and Chemistry of Amorphous LiO_2_‐ Containing Products in Li–O_2_ Batteries Using Cryogenic Electron Microscopy,” Journal of the American Chemical Society 144, no. 5 (2022): 2129–2136. 35075901 10.1021/jacs.1c10146

[154] S. Liu , Z. Hu , L. Zuo , et al., “Pt‐Doped CO_3_O_4_ Nanowires as High‐Efficiency Cathodic Catalysts for Improved Lithium‐Oxygen Batteries and Insights into the Electrochemical Reaction Pathway,” Electrochimica Acta 512 (2025): 145479.

[155] Y. Zhang , J. Ma , Z. Zhuang , et al., “Ordered Adsorption of Oxygen via High‐Density Low‐Coordinated Ru Sites for Lithium–Oxygen Battery,” ACS Catalysis 14, no. 24 (2024): 18851–18860.

[156] L.‐N. Song , L.‐J. Zheng , X.‐X. Wang , et al., “Aprotic Lithium–Oxygen Batteries Based on Nonsolid Discharge Products,” Journal of the American Chemical Society 146, no. 2 (2024): 1305–1317. 38169369 10.1021/jacs.3c08656

[157] Y. Li , Y. Qiu , Y. Wang , L. Wang , and Q. Lv , “Atomically Asymmetrical Ruthenium–Oxygen–Cobalt Sites Accelerate Oxygen Redox and Suppress Side Reactions for Stable Lithium–Oxygen Batteries,” ACS Nano 19, no. 36 (2025): 32643–32653. 40906901 10.1021/acsnano.5c10218

[158] S. Deng , K. Han , T. Wang , G. Sun , and H. Li , “Construction of a Mn‐Ti_3_C_2_ Three‐Dimensional Multifunctional Self‐Supporting Cathode for Lithium‐Oxygen Batteries,” Journal of Colloid and Interface Science 703 (2026): 139153. 41046615 10.1016/j.jcis.2025.139153

[159] X.‐Y. Yuan , D.‐H. Guan , X.‐X. Wang , J.‐Y. Li , C.‐L. Miao , and J.‐J. Xu , “A Magnetic Field‐Assisted Lithium‐Oxygen Batteries with Enhanced Reaction Kinetics by Spin‐Polarization Strategy,” Angewandte Chemie International Edition 64, no. 19 (2025): 202421361. 10.1002/anie.202421361 40022575

[160] M. Liu , Y. Gao , S. Guan , et al., “Harnessing Atomically Precise Fe‐Nx Motifs Within Hierarchical Porous Carbon‐MOF Matrices for Next‐Generation Ultrastable High‐Energy Lithium–Oxygen Batteries,” Carbon 247 (2026): 121069.

[161] Z. Zhang , M. Yang , K. Sun , and T. Wei , “Application of Highly Catalytic and Conductive Three‐Dimensional Carbon Network Structures in Lithium‐Oxygen Battery Cathodes,” Electrochimica Acta 568 (2026): 148939.

[162] P. Zhang , Z. Wang , P. Wang , et al., “Heteroatom Doping‐Induced Defected Co_3_O_4_ Electrode for High‐Performance Lithium Oxygen Battery,” ACS Applied Energy Materials 5, no. 3 (2022): 3359–3368.

[163] Y. Zhang , S. Zhang , H. Li , et al., “Tunable Oxygen Vacancies of Cobalt Oxides in Lithium–Oxygen Batteries: Morphology Control of Discharge Product,” Nano Letters 23, no. 19 (2023): 9119–9125. 37773017 10.1021/acs.nanolett.3c03025

[164] Y. Su , Z. Zhao , E. Wang , and Z. Peng , “Mechanistic Study on Oxygen Reduction Reaction in High‐Concentrated Electrolytes for Aprotic Lithium–Oxygen Batteries,” The Journal of Physical Chemistry Letters 15, no. 4 (2024): 10111–10117. 39331821 10.1021/acs.jpclett.4c02455

[165] H.‐M. Guan , Z.‐P. Cai , X.‐Y. Wu , K.‐X. Wang , and J.‐S. Chen , “Unlocking the Power of Lewis Basicity in Oxide Lattice Oxygens: A Regulating Force for Enhanced Oxygen Evolution Kinetics in Li‐O_2_ Batteries,” Angewandte Chemie International Edition 64, no. 35 (2025): 202509132. 10.1002/anie.202509132 40605280

[166] Y. Pan , A. Hu , R. Xu , et al., “Modulating Coordination Environment of Cobalt‐Based Spinel Octahedral Metal Sites to Boost Metal–Oxygen Bond Covalency for Reversible Lithium–Oxygen Batteries,” ACS Sustainable Chemistry & Engineering 12, no. 47 (2024): 17177–17189.

[167] Z. Wu , C. Yan , P. Gao , et al., “Redox Couple Strategy for Improving the Oxygen Redox Activity and Reversibility of Li‐ and Mn‐Rich Cathode Materials,” Nano Letters 24, no. 43 (2024): 13496–13503. 39412214 10.1021/acs.nanolett.4c02588

[168] M. Xie , N. Wang , S. Zhao , Z. Li , Y. Lu , and Q. Liu , “Optimizing the Adsorption of Intermediates through Modulating Mn D‐Band Centers by the Mn‐Ce Heterojunction for High‐Performance Lithium‐Oxygen Batteries,” Journal of Power Sources 605 (2024): 234512.

[169] Y. Wang , R. Wang , and Y. Li , “Atomically dispersed Transition Metal‐N_4_ Doped Graphene as a LiO Nucleation Site in Nonaqueous Lithium‐Oxygen Batteries,” Electrochimica Acta 422 (2022): 140554.

[170] J. Li , P. Sanglier , H. Qu , Z. Miao , Y. Li , and B.‐L. Su , “Photo‐Assisted Lithium–Oxygen Batteries: Mechanisms, Materials, and Perspectives,” Advanced Materials 38, no. 9 (2026): 18355. 10.1002/adma.202518355 41431435

[171] H. Shabbir , A. Butt , C.‐C. Yang , and R. Jose , “Catalysts‐Redox Mediators‐Interfaces Synergies in Aprotic Lithium Oxygen Batteries: Pathways to Stability and Efficiency,” Small 22, no. 11 (2026): 12962. 10.1002/smll.202512962 41504216

[172] Z. Zhang , D. Huang , X. Peng , Z. Zhang , Y. Dou , and Z. Zhou , “Surface Properties of Electrode Materials: A Key Factor Affecting the Catalytic Activity of Redox Mediators in Li– O_2_ Battery Discharge,” Energy & Environmental Materials 9, no. 1 (2026): 70107.

[173] K. Kannari , A. Ge , C. Xu , K.‐I. Inoue , and S. Ye , “From Bulk to Surface: A Raman Spectroscopic Analysis of Solvation Structures in Concentrated Acetonitrile Electrolytes for Li–O_2_ Batteries,” The Journal of Physical Chemistry C 128, no. 47 (2024): 20148–20155.

[174] S. T. Plunkett , A. Kondori , D. Y. Chung , et al., “A New Cathode Material for a Li–O_2_ Battery Based on Lithium Superoxide,” ACS Energy Letters 7, no. 8 (2022): 2619–2626.

[175] J. Ye , C. Wang , Z. Shao , and K. Liao , “From Li–O_2_ to Li–Air Batteries: Challenges and Progress on Oxygen‐Permeable Membranes,” Energy & Fuels 38, no. 16 (2024): 15001–15024.

[176] K. X. Wang , Q. C. Zhu , and J. S. Chen , “Strategies toward High‐Performance Cathode Materials for Lithium–Oxygen Batteries,” Small 14, no. 27 (2018): 1800078. 10.1002/smll.201800078 29750439

[177] L. Ma , T. Yu , E. Tzoganakis , et al., “Fundamental Understanding and Material Challenges in Rechargeable Nonaqueous Li–O_2_ Batteries: Recent Progress and Perspective,” Advanced Energy Materials 8, no. 22 (2018): 1800348.

[178] A. Sarabandi , A. Adam , and X. Li , “Influence of Electrolyte Saturation on the Performance of Li–O_2_ Batteries,” ACS Applied Materials & Interfaces 16, no. 45 (2024): 62902–62913. 39494669 10.1021/acsami.4c12168

[179] L. Dai , X. Zhou , Y. Yang , P. Hu , and L. Ci , “Ordered Porous Mn−Co Spinel Oxide (CoMn_2_O_4_) With Vacancies Modulation as Efficient Electrocatalyst for Li−O_2_ Battery,” Journal of Colloid and Interface Science 670 (2024): 719–728. 38788439 10.1016/j.jcis.2024.05.144

[180] J. Wang , R. Gao , D. Zhou , et al., “Boosting the Electrocatalytic Activity of Co_3_O_4_ Nanosheets for a Li‐O_2_ Battery through Modulating Inner Oxygen Vacancy and Exterior Co^3+^/Co^2+^ Ratio,” ACS Catalysis 7, no. 10 (2017): 6533–6541.

[181] L. Dai , Q. Sun , L. Chen , et al., “Ag Doped Urchin‐Like α‐MnO_2_ Toward Efficient and Bifunctional Electrocatalysts for Li‐O_2_ Batteries,” Nano Research 13, no. 9 (2020): 2356–2364.

[182] P. Zhang , R. Wang , M. He , J. Lang , S. Xu , and X. Yan , “3D Hierarchical Co/CoO‐Graphene‐Carbonized Melamine Foam as a Superior Cathode toward Long‐Life Lithium Oxygen Batteries,” Advanced Functional Materials 26, no. 9 (2016): 1354–1364.

[183] H. Wu , W. Sun , J. Shen , et al., “Improved Structural Design of Single‐ and Double‐Wall MnCo_2_O_4_ Nanotube Cathodes for Long‐Life Li–O_2_ Batteries,” Nanoscale 10, no. 27 (2018): 13149–13158. 29963679 10.1039/c8nr02795a

[184] L. M. Leng , X. Y. Zeng , H. Y. Song , T. Shu , H. S. Wang , and S. J. Liao , “Pd Nanoparticles Decorating Flower‐Like Co_3_O_4_ Nanowire Clusters to Form an Efficient, Carbon/Binder‐Free Cathode for Li–O_2_ Batteries,” Journal of Materials Chemistry A 3, no. 30 (2015): 15626–15632.

[185] X. Lin , L. Zhou , T. Huang , and A. Yu , “Hierarchically Porous Honeycomb‐Like Carbon as a Lithium–Oxygen Electrode,” Journal of Materials Chemistry A 1, no. 4 (2013): 1239–1245.

[186] X. Han , F. Cheng , C. Chen , Y. Hu , and J. Chen , “Uniform MnO_2_ Nanostructures Supported on Hierarchically Porous Carbon as Efficient Electrocatalysts for Rechargeable Li‐O_2_ Batteries,” Nano Research 8, no. 1 (2015): 156–164.

[187] X. Lin , R. Yuan , S. Cai , et al., “An Open‐Structured Matrix as Oxygen Cathode with High Catalytic Activity and Large Li_2_O_2_ Accommodations for Lithium–Oxygen Batteries,” Advanced Energy Materials 8, no. 18 (2018): 1800089.

[188] M. Zheng , J. Jiang , Z. Lin , P. He , Y. Shi , and H. Zhou , “Stable Voltage Cutoff Cycle Cathode with Tunable and Ordered Porous Structure for Li‐O 2 Batteries,” Small 14 (2018): 1803607. 10.1002/smll.201803607 30318700

[189] L. Cao , B. Zhang , X. Ou , C. Wang , C. Peng , and J. Zhang , “Interlayer Expanded SnS_2_ Anchored on Nitrogen‐Doped Graphene Nanosheets with Enhanced Potassium Storage,” ChemElectroChem 6, no. 8 (2019): 2254–2263.

[190] H. Tan , D. Chen , X. Rui , and Y. Yu , “Peering into Alloy Anodes for Sodium‐Ion Batteries: Current Trends, Challenges, and Opportunities,” Advanced Functional Materials 29, no. 14 (2019): 1808745.

[191] J. B. Varley , V. Viswanathan , J. K. Norskov , and A. C. Luntz , “Lithium and Oxygen Vacancies and Their Role in Li_2_O_2_ Charge Transport in Li–O_2_ Batteries,” Energy & Environmental Science 7, no. 2 (2014): 720–727.

[192] Y. Wang , P. Han , X. Lv , L. Zhang , and G. Zheng , “Reduction,” Joule 2, no. 12 (2018): 2551–2582.

[193] J. Li , S. Zhang , G. Zeng , et al., “Lif‐Induced Formation of Quartz Nanodomains in Micron‐Sized SiO_x_ Anodes for Durable Lithium‐Ion Batteries,” ACS Applied Energy Materials 8, no. 11 (2025): 7753–7761.

[194] G. Zeng , Q. Sun , S. Horta , et al., “Modulating the Solvation Structure to Enhance Amorphous Solid Electrolyte Interface Formation for Ultra‐Stable Aqueous Zinc Anode,” Energy & Environmental Science 18, no. 4 (2025): 1683–1695.

[195] Q. Sun , G. Zeng , X. Xu , et al., “Are Sulfide‐Based Solid‐State Electrolytes the Best Pair for Si Anodes in Li‐Ion Batteries?,” Advanced Energy Materials 14, no. 40 (2024): 2402048.

[196] J. Li , J. Guo , Q. Sun , et al., “Potassium Ions Regulated the Disproportionation of Silicon Monoxide Boosting Its Performance for Lithium‐Ion Battery Anodes,” Energy & Fuels 35, no. 19 (2021): 16202–16211.

[197] J. Guo , W. Zhai , Q. Sun , et al., “Facilely Tunable Core‐Shell Si@SiO_x_ Nanostructures Prepared in Aqueous Solution for Lithium Ion Battery Anode,” Electrochimica Acta 342 (2020): 136068.

[198] S. Wang , M. Xie , J. Wen , et al., “CeO_2_ Regulated Vacancies and Coordination Environment of Δ‐MnO_2_ Cathode for Durable Flexible Zinc‐Ion Batteries,” Journal of Energy Chemistry 116 (2026): 482–493.

[199] Y. Zhang , L. Tao , C. Xie , et al., “Defect Engineering on Electrode Materials for Rechargeable Batteries,” Advanced Materials 32, no. 7 (2020): 1905923. 10.1002/adma.201905923 31930593

[200] L. Xu , Q. Jiang , Z. Xiao , et al., “Plasma‐Engraved Co_3_O_4_ Nanosheets with Oxygen Vacancies and High Surface Area for the Oxygen Evolution Reaction,” Angewandte Chemie International Edition 55, no. 17 (2016): 5277–5281. 26990905 10.1002/anie.201600687

[201] Y. Wang , T. Zhou , K. Jiang , et al., “Reduced Mesoporous Co_3_O_4_ Nanowires as Efficient Water Oxidation Electrocatalysts and Supercapacitor Electrodes,” Advanced Energy Materials 4, no. 16 (2014): 1400696.

[202] X. Liu , L. Zhao , H. Xu , et al., “Tunable Cationic Vacancies of Cobalt Oxides for Efficient Electrocatalysis in Li–O_2_ Batteries,” Advanced Energy Materials 10, no. 40 (2020): 2001415.

[203] J. Li , C. Shu , A. Hu , et al., “Tuning Oxygen Non‐Stoichiometric Surface via Defect Engineering to Promote the Catalysis Activity of Co_3_O_4_ in Li‐O_2_ Batteries,” Chemical Engineering Journal 381 (2020): 122678.

[204] Y. Cao , Z. Wei , J. He , et al., “α‐MnO2 Nanorods Grown In Situ on Graphene as Catalysts for Li–O_2_ Batteries With Excellent Electrochemical Performance,” Energy & Environmental Science 5, no. 12 (2012): 9765.

[205] C. Luo , H. Sun , Z. Jiang , et al., “Electrocatalysts of Mn and Ru Oxides Loaded on MWCNTs With 3D Structure and Synergistic Effect for Rechargeable Li‐O_2_ Battery,” Electrochimica Acta 282 (2018): 56–63.

[206] M. Wei , B. Li , C. Jin , et al., “A 3D Free‐Standing Thin Film Based on N, P‐Codoped Hollow Carbon Fibers Embedded with MOP Quantum Dots as High Efficient Oxygen Electrode for Li‐O_2_ Batteries,” Energy Storage Materials 17 (2019): 226–233.

[207] A. Hu , J. Long , C. Shu , et al., “Three‐Dimensional CoNi_2_S_4_ Nanorod Arrays Anchored on Carbon Textiles as an Integrated Cathode for High‐Rate and Long‐Life Lithium−Oxygen Battery,” Electrochimica Acta 301 (2019): 69–79.

[208] X.‐Y. Yang , J.‐J. Xu , Z.‐W. Chang , et al., “Blood‐Capillary‐Inspired, Free‐Standing, Flexible, and Low‐Cost Super‐Hydrophobic N‐CNTs@SS Cathodes for High‐Capacity, High‐Rate, and Stable Li‐Air Batteries,” Advanced Energy Materials 8, no. 12 (2018): 1702242.

[209] Z. Li , C. Song , P. Dai , et al., “Nonvolatile and Nonflammable Sulfolane‐Based Electrolyte Achieving Effective and Safe Operation of the Li–O_2_ Battery in Open O_2_ Environment,” Nano Letters 22, no. 2 (2022): 815–821. 34994574 10.1021/acs.nanolett.1c04537

[210] B. Wang , C. Liu , L. Yang , Q. Wu , X. Wang , and Z. Hu , “Defect‐Induced Deposition of Manganese Oxides on Hierarchical Carbon Nanocages for High‐Performance Lithium‐Oxygen Batteries,” Nano Research 15 (2022): 4132–4136.

[211] S. Deshmukh , S. Nouseen , and M. Pumera , “Laser‐Induced Microfabrication of Carbon Nanostructure: Processing Mechanism and Application for Next‐Generation Battery Technology,” Advanced Functional Materials 36, no. 19 (2026): 19979.

[212] A. U. Ahmad , W. G. Morais , T. Ahmad , et al., “In Situ Growth of Mixed‐Valence 2D α‐MnO_x_ Nanosheets Within Interlayer Spaces of Multilayer Ti_3_C_2_ MXene as an Efficient Air Cathode for Rechargeable Li–O_2_ Batteries,” Journal of Materials Chemistry A 14, no. 29 (2026): 18571–18577.

